# A 3D system to model human pancreas development and its reference single-cell transcriptome atlas identify signaling pathways required for progenitor expansion

**DOI:** 10.1038/s41467-021-23295-6

**Published:** 2021-05-25

**Authors:** Carla A. Gonçalves, Michael Larsen, Sascha Jung, Johannes Stratmann, Akiko Nakamura, Marit Leuschner, Lena Hersemann, Rashmiparvathi Keshara, Signe Perlman, Lene Lundvall, Lea Langhoff Thuesen, Kristine Juul Hare, Ido Amit, Anne Jørgensen, Yung Hae Kim, Antonio del Sol, Anne Grapin-Botton

**Affiliations:** 1 The Novo Nordisk Foundation Center for Stem Cell Biology, Copenhagen, Denmark; 2 CIC bioGUNE-BRTA (Basque Research and Technology Alliance), Derio, Spain; 3 Max Planck Institute of Molecular Cell Biology and Genetics, Dresden, Germany; 4 Department of Gynaecology, University Hospital of Copenhagen (Rigshospitalet), Copenhagen, Denmark; 5 Department of Obstetrics and Gynaecology, Hvidovre University Hospital, Hvidovre, Denmark; 6 The Weizmann institute, Rehovot, Israel; 7 Department of Growth and Reproduction, Copenhagen University Hospital (Righshospitalet), Copenhagen, Denmark; 8 Luxembourg Centre for Systems Biomedicine (LCSB), University of Luxembourg, Belvaux, Luxembourg; 9 IKERBASQUE, Basque Foundation for Science, Bilbao, Spain

**Keywords:** Organogenesis, Stem-cell differentiation

## Abstract

Human organogenesis remains relatively unexplored for ethical and practical reasons. Here, we report the establishment of a single-cell transcriptome atlas of the human fetal pancreas between 7 and 10 post-conceptional weeks of development. To interrogate cell–cell interactions, we describe InterCom, an R-Package we developed for identifying receptor–ligand pairs and their downstream effects. We further report the establishment of a human pancreas culture system starting from fetal tissue or human pluripotent stem cells, enabling the long-term maintenance of pancreas progenitors in a minimal, defined medium in three-dimensions. Benchmarking the cells produced in 2-dimensions and those expanded in 3-dimensions to fetal tissue identifies that progenitors expanded in 3-dimensions are transcriptionally closer to the fetal pancreas. We further demonstrate the potential of this system as a screening platform and identify the importance of the EGF and FGF pathways controlling human pancreas progenitor expansion.

## Abstract (web summary)

From single-cell transcriptome analyses to defining culture media for spheroids, the authors provide a census of information to understand the development of human pancreatic progenitors. This approach identifies signalling pathways (EGF and FGF) regulating progenitor proliferation.

## Introduction

Human pancreatic progenitors first appear at the foregut–midgut boundary at around 30 days post conception (dpc), marked by the key transcription factor pancreatic and duodenal homeobox factor 1 (PDX1). These cells are highly proliferative, and by analogy to the mouse pancreas, it is assumed that they are multipotent and can thus give rise to all cell types within the adult pancreas. Among those, acinar and ductal cells are responsible for the production and secretion of digestive enzymes and make up most of the adult pancreas. The islets of Langerhans make up 1 to 2% of the organ and encompass multiple cell types, including the β-cells, which are crucial to maintain glucose homeostasis. Multiple mutations have been described to cause developmental defects resulting in pancreatic dysfunction, such as neonatal diabetes and cystic dysplasia^1^. Some studies also suggest that type 2 diabetes has a developmental origin or predisposition although it appears later in life^2,3^. Despite the limited availability of human fetal pancreas samples, histological studies revealed the main milestones in human pancreas development to be similar to those observed in mouse^4^, while also pointing to important differences, such as a later onset of endocrine differentiation, β-cells appearing first in contrast to α-cells in rodents^5^, and a different islet morphology^6^. More recently, important transcriptomic data have been generated, ranging from bulk RNA sequencing of the pancreatic bud before progenitor specification^7,8^ to sequencing selected populations over weeks of development^9,10^. Single-cell transcriptomic data is available in mice, at a later developmental stage in human^11^ and in the adult^12–14^.

Recapitulating pancreas development in vitro has also been of high interest, with the major incentive being the potential of using β-cells for replacement therapies to treat diabetes. Several protocols were developed to differentiate β cells from human pluripotent stem cells (hPSC)^15–19^. These in vitro systems are useful to complement descriptive studies on isolated human fetal pancreas. Moreover, pancreatic organoids or spheres derived from hPSC cells are another tool to study human pancreas development, but so far have not been compared directly to the human fetal pancreas^20–23^.

Here we present a single-cell transcriptome dataset spanning fetal development from 7 to 10 weeks post conception (wpc), which we use as a benchmark for a new pancreatic progenitor culture system. While most human embryonic stem cell (hESC)-derived pancreas differentiation methods have been focused on the production of β cells for diabetes therapy, our aim was to trap progenitors in vitro in a state as close as possible to in vivo, rather than forcing their differentiation in non-physiological proportions and times. This system allows for self-organization and long-term expansion of pancreatic progenitors. Owing to the robustness achieved with this method, we develop it to perform small molecule screening and identify the minimum requirements needed for the self-renewal of human pancreatic progenitors in vitro.

## Results

### A single-cell transcriptome atlas of the fetal pancreas

**Fig. 1:**
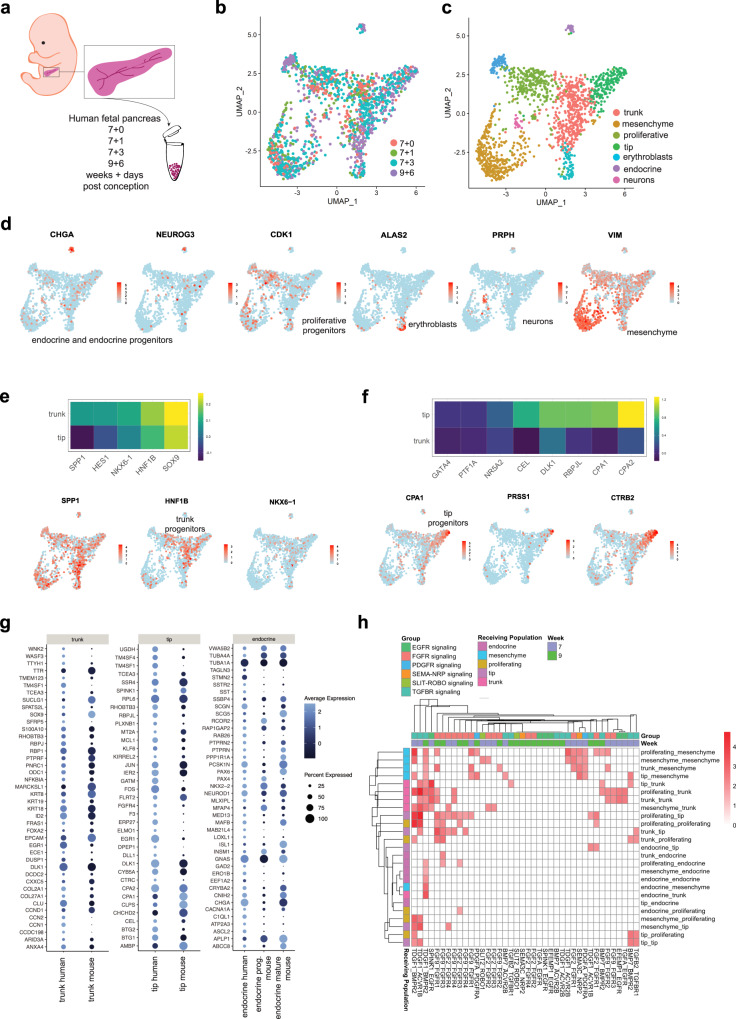
A single-cell transcriptome atlas of the fetal pancreas. **a** Whole pancreas was isolated from fetuses between 7 and 10 weeks post conception (wpc) and most of the mesenchyme was removed manually (*n* = 4). **b** UMAP of the entire fetal dataset colored according to the developmental stage of each sample (e.g., 7 weeks + 0 days). **c** UMAP of the entire fetal dataset colored according to the cluster identity determined through unsupervised clustering. We annotated 7 of the clusters according to their gene expression. The cell identity of the eighth cluster (dark blue) was uncertain. **d** Representative expression and distribution of known marker genes for endocrine cells, proliferating progenitors, erythroblasts, neurons, and mesenchyme. **e**, **f** Global average counts in trunk (**e**) and tip progenitors (**f**) for typical respective progenitor markers; Representative expression and distribution of selected genes enriched in trunk or tip progenitors. **g** Dot plot representing gene expression differences between mouse and human in three equivalent populations. The selected genes are those most highly specific for each population. **h** Heatmap representing the most likely ligand (first name)–receptor (second name) interactions (likelihood score based on abundance) at different stages and in different population pairs (first ligand source, second receptor population). See also Supplementary Data 1 and Supplementary Data 2. See also Supplementary Fig. 1.

We have previously reported on transcriptional profiling of sorted bulk populations isolated from the human fetal pancreas, which included progenitors, endocrine progenitors, and endocrine cells^9^. We have also studied their single-cell expression profile (EP) by single-cell qPCR^24^. Here we used MARS-seq^25^ to transcriptionally profile 1465 cells between 7 and 9 + 6 wpc (Fig. 1a–c). We were able to map the main cell populations based on their EP. We identified a prominent population of mesenchymal cells marked by broad expression of *VIM*, *DCN*, and multiple collagens. The other main population was composed of progenitors with broad expression of *PDX1*, *SOX9*, and *KRT19*. We also identified a small (1.8%) population of endocrine cells, marked by expression of *CHGA, ISL1*, and *INS*. In agreement with previous reports on the delayed emergence of α-cells in human, *GCG* was not detected in this population^26,27^. Non-pancreatic cell types such as neurons (*GAP43*, *PRPH*) and red blood cells (*ALAS2*, *HBG1*) were also present (Fig. 1d). Among the pancreatic progenitor population, three separate EPs were found. The first and most abundant cell type corresponded to a population similar to mouse trunk progenitors^28,29^ with higher expression of *SOX9*, *HNF1B, HES1*, and *NKX6-1* (Fig. 1e, Supplementary Fig. 1). Interestingly, *SPP1*, a known ductal marker in the adult human pancreas^30^, was also found to be increased in this population. The second group had a very similar EP to the trunk progenitors but in addition had increased levels of CDK1 and PCNA, among other markers which suggested actively proliferating cells (Fig. 1d). Lastly, we found cells where *CPA1*, *RBPJL*, and *CEL* were highly expressed (Fig. 1f), suggesting a population similar to the mouse tip progenitors^31^. A few cells within the tip progenitor population also expressed *PRSS1*, indicating they may be early acinar cells. We also found *CTRB2* to be highly specific for this population. A list containing many additional markers for each population is provided in Supplementary Data 1, and several known markers are mapped on the UMAP in Supplementary Fig. 1c.

To evaluate whether there were differences between mouse and human EPs, we compared our dataset to a previously reported dataset containing all pancreatic cell types at a comparable stage of development^32^. This comparison revealed similar populations in the mouse and human datasets (Supplementary Fig. 1a). For each population we established the 50 most variable genes between different human populations, enabling to discriminate each of them. We then explored whether their expression would be similar in mouse (Fig. 1g). While most genes were found to mark a similar proportion of cells and had a comparable abundance per cell, we observed some differences. Notably, *SOX9*, *KRT8*, and *SUCLG1* were expressed in more cells and at higher levels in mouse, while the opposite was seen for *RBPJ, EGR1*, and *TM4SF1* in the trunk progenitors (Fig. 1g). In the tip progenitors, *RBPJL*, *TM4SF1* and *4*, *GATM*, *EGR1*, *FGFR4*, and *FOS* were expressed in more cells and at higher level in human. Moreover, while *CPA1* was enriched in mouse, *CPA2* was enriched in human (Fig. 1g). With regards to human endocrine cells, the cells we captured had a signature with similarities to both mouse endocrine progenitors and endocrine cells (Fig. 1g). Qualitatively similar results were obtained when including an additional mouse dataset from Bastidas-Ponce et al.^33^.

In addition to revealing individual gene expression in these populations, we investigated the functional cell–cell communication events between the different cell types. To do so, we developed InterCom, a mechanistic computational model that integrates extracellular binding events with intracellular signaling cascades and transcriptional regulation. Employing a collection of manually curated ligand–receptor interactions, InterCom assigns a score to each interaction that considers the average expression of both molecules and assesses its significance. Multiple interactions between matrix and integrins as well as other matrix receptors such as DDR1^34^ exhibited the highest strength scores (Supplementary Data 2 for a global list). In addition, we identified interactions via the TGFβ pathway involving *BMP7* in epithelium and *BMPR2* in mesenchyme/epithelium, as previously observed in mice^35,36^, as well as the *TGFB2/TGFBR1* pair within the epithelium (Fig. 1h, Supplementary Data 2). As in mice, the FGF pathway appeared to signal with both ligands and the four receptors in epithelium and mesenchyme. A notable difference was that while FGF10 and 7 are the ligands reported in mice, *FGF2* and *9* were the most abundant in human at these stages (Fig. 1h, Supplementary Fig. 1b, Supplementary Data 2). The EGF pathway is also known to operate during mouse pancreas development, and it was also detected in our analysis. *EGFR* was present in the human fetal epithelium, but the EFGR ligands we detected in human, namely *TGFA*, *EFEMP1/FBLN3*^37^, and another possible ligand *SPINK1*^38^, were different from those reported previously in mouse^39^ (Fig. 1h, Supplementary Fig. 1b, Supplementary Data 2). In addition, we found that *PDGFA* was produced in the epithelium and its receptor *PDGFRA* was detected in the mesenchyme (Fig. 1h, Supplementary Fig. 1b, Supplementary Data 2). Axon guidance pathways including SLIT2-ROBO1, which have recently been investigated in the mouse pancreas^40–43^, and SEMA3C-NRP2, which has not, were also predicted to signal to the mesenchyme (Fig. 1h). In comparison, state-of-the-art tools for cell–cell communication analysis were largely not able to recapitulate these interactions (Supplementary Note 1, Supplementary Data 3). However, they complement Intercom’s interactions, which are by design restricted to secreted ligands, with cell–cell contact based interactions involving, for instance, Notch signaling.

### A 3D long-term culture system of human pancreatic progenitors

**Fig. 2:**
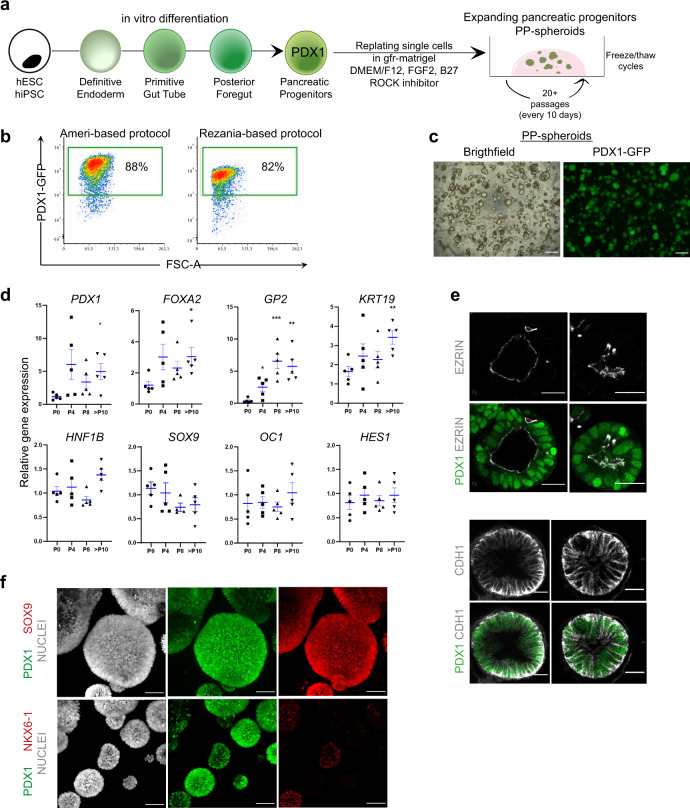
A minimal culture system allowing for long-term culture of human spheroids harboring pancreatic progenitors. **a** Starting from hiPSC or hESC lines, we performed stepwise differentiation until the pancreatic progenitor stage (S4d8 for Ameri protocol and S4d3 for Rezania protocol, PDX1+), at which point cells were re-plated in 3D Matrigel with FGF2, B27, and ROCK inhibitor Y-27632 supplementation. In these conditions, PP-spheroids formed and could be passaged every 10 days for at least 20 passages, including going through freeze/thaw cycles. **b** Prior to re-plating, the percentage of PDX1 cells was measured to ensure a successful differentiation. Representative intracellular flow cytometry analysis for PDX1 at the end of stage 4 of the protocol based on Ameri et al.^45^ and the protocol based on Rezania et al.^16^. **c** PP-spheroids at day 10 in culture, scale bar = 250 µm. **d** Expression of pancreatic progenitor genes in PP-spheroids at different passages (*N* = 5), data shown as mean ± SEM. *P < 0.05, **P < 0.01, ***P < 0.001. *P* values were determined by two-sided Mann–Whitney test. **e** Optical section of whole-mount images showing PDX1, EZRIN, and CDH1 staining, scale bar = 25 µm. **f** Z-stack projection of whole-mount images showing PDX1, SOX9, and NKX6-1 staining, scale bar = 50 µm. Images shown in **c**, **e**, and **f** are representative of over ten independent experiments. See also Supplementary Fig. 2.

To complement these static observations, we developed a culture system to enable the study of human pancreas development in three dimensions. In vitro models of human pancreas development have been optimized for the production of β cells for the purpose of cell replacement therapies for diabetes. We took advantage of such protocols to produce pancreas progenitors in two-dimensional (2D) cultures, starting from hESC or induced pluripotent stem cell (iPSC) lines. Such progenitors were then re-plated in three-dimensional (3D) conditions in Matrigel, with a culture medium containing FGF2, B27, and ROCK inhibitor (Y-27632), based on a culture medium we previously designed for the 3D culture of mouse pancreas progenitors^44^ (Fig. 2a). We used two previously published differentiation protocols which reliably generated high percentages of PDX1+ cells at the end of the pancreatic progenitor specification stage^16,45^ (Fig. 2b). Pancreatic progenitors captured in this way have been shown to be amenable to expansion when co-cultured with a mouse embryonic fibroblast cell line^46^.

In our 3D culture conditions, pancreatic progenitors derived with either protocol formed spheres and spheroids (globally referred to as PP-spheroids thereafter) that could subsequently be passaged every 10 days (Fig. 2c). Pancreatic progenitor markers *SOX9*, *HNF1B*, *HES1*, and *ONECUT1* were stably expressed, while a modest increase of the progenitor markers *PDX1* and *FOXA2* as well as the progenitor/ductal marker *KRT19* was observed, particularly in PP-spheroids passaged over ten times. We also observed a steady increase in *GP2*, a surface marker expressed in human multipotent progenitors^10^ (Fig. 2d). The endocrine and trunk progenitor marker *NKX6-1* was lowly expressed and even undetectable in 6 out of 20 samples tested (Supplementary Fig. 2a). While PDX1, SOX9, and CDH1 were homogeneously expressed, NKX6-1 was heterogeneous (Fig. 2e, f). Morphologically, we detected both spheres with cells organized around a large central lumen and spheroids with small lumens that formed partially connected networks over time (Fig. 2e). We found the presented expansion culture method to be robust across multiple hESCs (HuES4, H1, and H9) and iPSC (SBAD3.1 and SBAD3.4) lines. In addition, we found it suitable for primary culture of dissociated cells from the human fetal pancreas (fetal spheres). We were able to expand fetal spheres for at least three passages and observed that non-endoderm cells (e.g., mesenchymal) were progressively depleted (Supplementary Fig. 2d), while progenitor marker expression was preserved (Supplementary Fig. 2b, c). Here, as in hESC-derived objects, we observed both spheres with a large lumen (Supplementary Fig. 2d, passage 1) and spheroids with a network of thin lumen (Supplementary Fig. 2c).

### Benchmarking of cells in vitro to human fetal pancreas

**Fig. 3:**
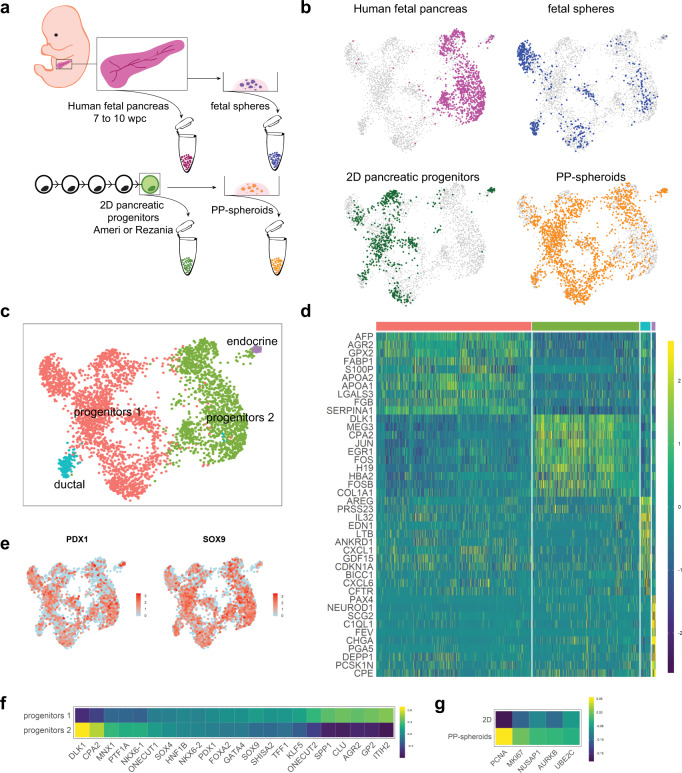
Benchmarking of cells in vitro to human fetal pancreas. **a** In addition to fetal pancreas samples, we sequenced fetal spheres which are fetal pancreas cells cultured in expansion conditions; 2D pancreatic progenitors which are hPSCs differentiated with one of two protocols; and PP-spheroids which are hPSC differentiated in vitro and cultured in 3D in expansion conditions (HUES4, H1, SBAD3.4). **b** UMAP plots of the overall data after removing non-epithelial cells (mesenchymal, neuronal, and blood cells). In each plot the cells are highlighted according to their known origin. **c** Cells are colored according to their cluster identity, assigned by unsupervised clustering. **d** A heatmap of the top ten genes enriched in each cluster. **e** UMAP showing broad expression of the pancreatic progenitor genes *PDX1* and *SOX9*. **f** Global average counts in progenitors 1 and progenitors 2 for selected pancreatic genes highlighting differences between the two populations. **g** Global average counts in PP-spheroids and 2D progenitors for selected genes associated with cell proliferation. See also Supplementary Fig. 3.

In order to assess the similarity of the cells expanded in culture to endogenous cells, we benchmarked the model by sequencing 2063 PP-spheroid cells and comparing them to the fetal pancreas dataset mentioned previously. In addition, to assess the effect of the 3D culture, we included 662 pancreas progenitors produced in 2D from hESCs/iPSCs. We also included 662 cells from the fetal spheres, to test the effect of the expansion medium directly on fetal cells. In order to restrict the comparison to pancreatic cell types, we excluded non-epithelial pancreatic cells before proceeding with the analysis (Fig. 3a, b). This included the mesenchymal, neural, and red blood cells from the fetal pancreas (Fig. 1), as well as a small proportion of mesenchyme arising from the fetal spheres (Supplementary Fig. 2d). Dimensionality reduction resulted in four major clusters. Two clusters had a progenitor-like EP (*SOX9*, *PDX1*), one appeared to be ductal (*CFTR*, *BICC1*), and the last was a cluster of cells expressing endocrine markers (*CHGA*, *NEUROD1, INS, GHRL*) (Fig. 3c–e). Strikingly, we found that human fetal pancreas samples and 2D progenitors segregated to different clusters (Fig. 3b). 2D cells fell mostly within ‘progenitors 1’ and ductal cell clusters, while cells of the human fetal pancreas were confined to ‘progenitors 2’ cluster (Fig. 3c). The exception was the small endocrine cluster, which contained cells from all samples (Fig. 3b, c). Notably, both the 3D-expanded PP-spheroids and fetal spheres were distributed across all four clusters, indicating that at least part of the progenitor cells comprising them had an EP that more closely resembled fetal pancreas progenitors. Early liver markers (*AFP*, *FABP1*, *APOL1* & 2, etc.) as well as the pancreas progenitor markers *GP2*, *SPP1*, and *ONECUT2* were enriched in ‘progenitors 1’ cluster containing the cells from 2D culture (Fig. 3c–f, Supplementary Fig. 3a). *DLK1*, *NKX6*-1, *MNX1*, and early response genes (*FOS*, *JUN*, *EGR1*) were increased in ‘progenitors 2’ cluster, enriched in cells freshly dissociated from the fetal pancreas (Fig. 3c–f).

A direct comparison of cells grown in 3D and in 2D, excluding all other populations gave more power in discriminating differences and showed that the cells in 2D had enriched liver markers (*TTR*, *AFP*, *APOE*), whereas the cells in 3D expressed more *GP2*, *AGR2*, *ANXA4*, and *SPP1* (Supplementary Fig. 3c). Notably, the liver markers found in 2D progenitors were expressed in cells that also expressed pancreatic markers, and the expression levels were high across all cells and higher than in any other conditions, including in vivo (Supplementary Fig. 3a). Another notable difference between 2D and 3D was the higher percentage of cells in G2/M in 3D conditions (3% in 2D compared to 15% in 3D), as estimated based on the expression of cell cycle annotated genes^47^. This is likely to be symptomatic of contact inhibition in 2D. We also observed that while cells produced using different protocols distributed similarly in the global UMAP, early spontaneous differentiation into the endocrine path was seen only in the Rezania et al.^16^ protocol (Supplementary Fig. 3g), likely a result of BMP inhibition^18,48^. The EPs of progenitors arising from either the Ameri et al.^45^ or the Rezania et al.^16^ protocols were overall very similar. A few differences were however identified when comparing only the two populations in 2D, the most notable being higher expression of *ID1* and *IGFBP7* in cells differentiated with the Ameri et al.^45^ protocol, and higher expression of the liver markers *FGB* and *APOC3* in cells differentiated with the Rezania et al.^16^ protocol (Supplementary Fig. 3f), possibly due to the lower expression of PDX1, which represses liver genes^49^. The resulting PP-spheroids produced from the two protocols converged to a similar EP (Supplementary Fig. 3d). In early passages, the presence of liver markers co-expressed with pancreatic markers was noted in PP-spheroids, although lower than in 2D culture. This suggests the presence of immature cells endowed with the potency to form multiple organs (Supplementary Fig. 3c, d). However, over time, these cells evolved to a stricter pancreatic fate as seen by the loss of *AFP*, *APOE*, and *APOA1* (Supplementary Fig. 3c).

Comparing fetal cells in vitro and in vivo, we observed that a subset of the progenitors diverged in vitro, acquiring either a more mature ductal signature (*CFTR*, *SPP1*, *SOX9*^*high*^) or a progenitor signature (*PDX1*, *SOX9*^low^) (Supplementary Fig. 3a, h). The latter population, similarly to PP-spheroids, had also higher *GP2* and *AGR2*. Immunocytochemistry further suggested heterogeneity based on the expression of CFTR in small hollow spheres with low PDX1 levels and MUC1 in hollow spheres with high PDX1 levels and in spheroids, lining complex networks of narrow lumen (Supplementary Fig. 3b).

### Physiologically relevant proliferation rates of PP-spheroids

**Fig. 4:**
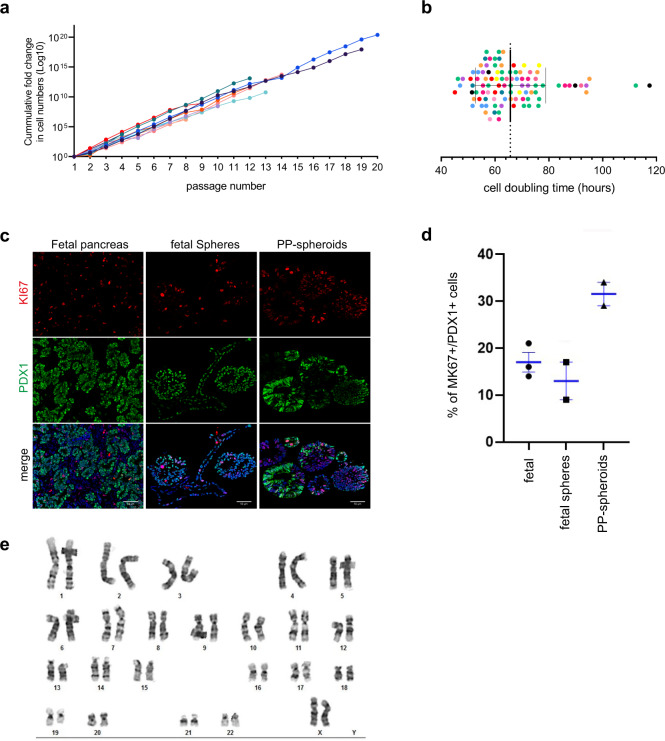
The conditions used for maintaining PP-spheroids are conducive of physiologically relevant proliferation rates. **a** Fold-change in cell number. Each line represents PP-spheroids generated independently from hPSC differentiation (H1, H9, HuES4, SBAD3.1, and SBAD3.4). Circles indicate each passaging point. The results are expressed as fold-change relative to the initial cell numbers. **b** Cell doubling calculated based on cells counted at the time of passaging. Each data point corresponds to a passaging event (*n* = 96). Colors represent independent expansion rounds (*N* = 11). **c** Representative tissue sections showing MKI67 and PDX1 in fetal pancreas, fetal spheres, and PP-spheroids. Scale bar = 50 µm. Images are representative of *N* = 3 fetal samples, *N* = 2 fetal spheres, *N* = 3 PP-sheroids. **d** The percentage of MKI67+cells over total number of PDX1+ cells. The samples are fetal pancreas at 7 + 5 wpc, 8 + 4 wpc, and 9 + 6 wpc (*N* = 3); fetal spheres at passage 1 and 3 (*N* = 2); PP-spheroids at passage 9 and 16 (*N* = 2) showing MKI67 and PDX1. Data shown as mean ± SEM. **e** Karyogram of H9 PP-spheroids at P5.

One of the bona-fide characteristics of progenitors is the ability to proliferate. We determined that in our culture system progenitors could be passaged at least 20 times, including freeze-thaw cycles, while stably proliferating (Fig. 4a). After around 20 passages, samples were stored, without indication that they could not be kept in culture indefinitely. During each passage, after 10 days in culture, PP-spheroids yielded a 15-fold expansion on average, which roughly represents a 65 h cell doubling time (Fig. 4b). This is comparable to previous reports of pancreatic progenitor proliferation in vitro^46^, although it likely underestimates proliferation rates, since it does not account for cell death upon re-plating.

We compared the levels of MKI67 in PP-spheroids, fetal spheres, and fetal pancreas to assess the relevance of proliferation in vitro as compared to in vivo and found a twofold increase in PP-spheroids relative to the other two samples (Fig. 4c, d). In fetal spheres, however, MKI67 levels were very similar to those found in vivo, indicating that the culture conditions are appropriately recapitulating physiological proliferation levels in pancreatic progenitors. The increase seen in PP-spheroids could simply be the result of genomic abnormality resulting in hyperproliferation, which is common among hPSC lines^50^. However, the karyotype of lines that were initially normal remained normal at P5 and P12 (Fig. 4e).

### Endocrine differentiation can be triggered in expanded PP-spheroids

Another hallmark of pancreas progenitors is their ability to differentiate. The pancreatic progenitors contained within PP-spheroids spontaneously differentiated into endocrine cells in the expansion medium, with a low efficiency, as evidenced by the presence of a small endocrine cluster (22 cells, 1.1% of all the PP-spheroid cells) (Supplementary Fig. 3d, e). However small, this percentage is not dissimilar to what is found in a human adult pancreas, with islets making up only 1–2% of the entire organ^51^. The percentage of endocrine cells seen in the fetal pancreas sequenced (20 cells, 1.8% of all the fetal cells excluding the mesenchyme) is also in accordance with this (Fig. 1c, d). Cells in the endocrine clusters expressed typical endocrine genes (*NEUROD1*, *INSULIN*, *CHRGA*, *NEUROG3*, *GHRL*, *FEV*, etc) (Fig. 3d).

**Fig. 5:**
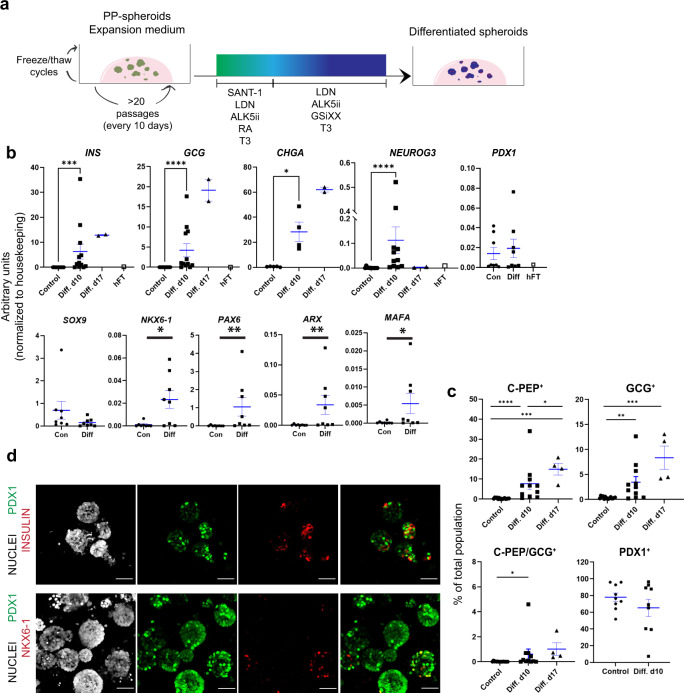
Endocrine differentiation can be triggered in expanded PP-spheroids. **a** Day 10 PP-spheroids (from HuES4, H9, H1, and SBAD3.4) in expansion medium for at least 2 passages after thawing were changed to a differentiation medium with two distinct stages (based on stages 5 (3 days) and 6 (7 or 14 days) of the Rezania et al.^16^ protocol) and analyzed after 10 days or 17 days. **b** Summary of qPCR analyses. Expression levels normalized to housekeeping genes are shown for PP-spheroids in expansion conditions (con) and after differentiation (diff). A fetal pancreas sample (9 + 3 wpc) was included as a control for some targets, *N* = 6–11, *N* = 2 for day 17. Data shown as mean ± SEM. *P < 0.05, **P < 0.01, ***P < 0.001, ****P < 0.0001. *P* values were determined by two-sided Mann–Whitney test. **c** Summary of flow cytometry analysis for C-PEPTIDE, GLUCAGON, and PDX1 in PP-spheroids before and after differentiation, *N* = 11 for control and day 10, *N* = 4 for day 17. Data shown as mean ± SEM. *P < 0.05, **P < 0.01, ***P < 0.001, ****P < 0.0001. *P* values were determined by a two-sided Mann–Whitney test. **d** Representative Z-stack projection of whole-mount images showing PDX1, INSULIN, and NKX6-1 in differentiated spheroids. Scale bar = 50 µm. Images are representative of *N* = 3 differentiation immunostainings. See also Supplementary Fig. 4.

We then challenged the expanded pancreatic progenitors to differentiate by subjecting PP-spheroids to stages 5 (3 days) and 6 (7 to 14 days) of the Rezania et al.^16^ protocol (Fig. 5a). At 7 days of stage 6, at day 10, we observed an increase in the expression of several endocrine genes (*NEUROG3*, *INS*, *GCG*, *NKX6*-1, *PAX4*, *ARX*, *MAFA*) and maintenance of *PDX1*, while *SOX9* was decreased (Fig. 5b). We quantified the numbers of C-PEPTIDE+ (a peptide cleaved from PRO-INSULIN) and GLUCAGON+ cells by flow cytometry, which showed higher percentages of C-PEPTIDE+ over GLUCAGON+ cells, and about 10% hormonal cells being bi-hormonal (Fig. 5C). Differentiation continued to progress between day 10 and 17 in stage 6 medium, reaching an average of 23% endocrine cells (Fig. 5b, c). We also confirmed that the number of PDX1+ cells remained close to what was observed in expanding PP-spheroids (Fig. 5c). Immunostainings revealed that, post treatment, most PP-spheroids contained a mixture of INSULIN+/PDX1 high cells, and PDX1+ cells. The levels of *NKX6-1* were also markedly higher than in expansion medium, although still heterogeneous (Fig. 5d). In summary, we can conclude that a significant portion of the cells within PP-spheroids behave like progenitors, and have the potential to progress to an endocrine fate. This was the case independently of the protocol used to differentiate pancreatic progenitors and across cell lines (HuES4, H1, H9 and SBAD3.4). Early (P3, P4, P5) and late (P12, P17) passage PP-spheroids were similarly responsive and increased expression of endocrine genes (Supplementary Fig. 4a). Despite a consistent shift toward the endocrine fate after treatment, we observed significant variations (Fig. 5b, c, Supplementary Fig. 4a). These were due primarily to a low differentiation capacity when passaged only once after thawing a batch, which was rescued at subsequent passages (compare Supplementary Fig. 4a, b, and Fig. 5c). Among cell lines, H1 and H9 were the most potent at differentiating (Supplementary Fig. 4a).

In order to further evaluate the potency of our expanded progenitors, we applied an exocrine differentiation protocol^52^ to PP-spheroids. We noted a significant increase in the expression of *PTF1A*, a known tip progenitor and acinar marker in mouse^31,53^, which has also been reported as a tip progenitor marker in human^11^. *CPA1* and *AMY2A* were also slightly increased, although not significantly (Supplementary Fig. 4c). However, the modest increase of these genes makes it unlikely that the cells became bona-fide acinar cells.

### Screening reveals control of PP proliferation by EGF and FGF pathways

**Fig. 6:**
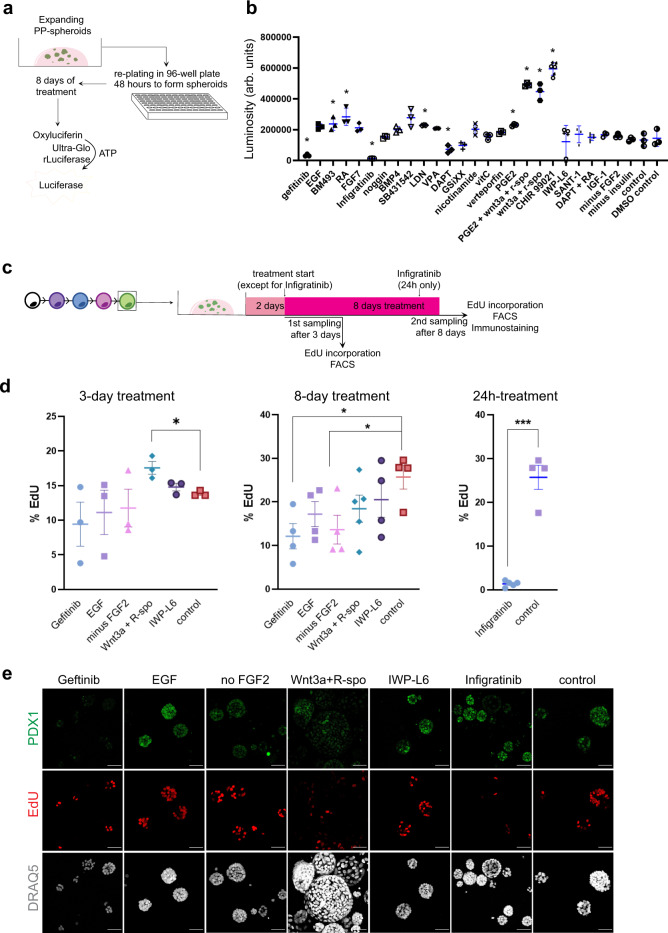
A developmental model system amenable to screening reveals the importance of EGF and FGF pathways in progenitor proliferation. **a** PP-spheroids (from HUES4) were passaged into a 96-well plate at normal density and treatments in different conditions started after 2 days. After 8 days of treatment, ATP in each well was measured with a Cell titer-Glo assay, as a proxy for cell numbers. **b** Luminosity (arb. units) reflects the amount of ATP in each well. Results shown as mean ± SD (*n* = 3). **c** For validation, we performed EdU quantification by flow cytometry (PP-spheroids from HUES4 and SBAD3.4) or whole-mount immunostaining (fetal spheres) after 3 or 8 days of treatment. Treatment times were identical to the screening except for Infigratinib which was used for the last 24 h only. **d** EdU percentages for different conditions after 3, 8, or 1 (for Infigratinib) day of treatment, *N* = 3. Data shown as mean ± SEM. *P < 0.05. *P* values were determined by a two-sided Mann–Whitney test. **e** Representative Z-stack projections of whole-mount images showing PDX1, EdU, and DRAQ5 (nuclear stain) in PP-spheroids treated with different conditions. Images are representative of *N* = 4 validation immunostainings. Scale bar = 50 µm. See also Supplementary Fig. 5.

The 3D culture system has the remarkable ability of maintaining pancreas progenitors in a proliferative, fetal-like state over long periods of time. It could therefore be a valuable in vitro platform to investigate the mechanisms of human pancreas development by doing interference experiments. The ability to produce large numbers of progenitors from PSCs and the high reproducibility of the expansion culture system make large scale screening possible. As a proof-of-concept, we selected several pathways known to have a prominent role in pancreas development, either in mouse or human, and tested how their modulation affected PP-spheroid proliferation. When possible we applied both activators and inhibitors for a given pathway. We allowed for passaged cells to form PP-spheroids for 48 h before starting the treatments. They were then treated for 8 days, keeping the experiment within the standard 10 days of culture (Fig. 6a). As a first readout, we measured the level of ATP in each well as a proxy for cell number. The assay was of high performance, with an excellent Z-factor (Z′ = 0.8), and a high signal to background ratio (s/b = 4.5). A second readout was an endocrine differentiation assay based on qPCR for *CHGA* run in a parallel plate. The most striking result was the sharp reduction in cell numbers after treatment with the EGFR inhibitor Gefitinib and the FGFR1/2/3 inhibitor Infigratinib (Fig. 6b). However, the addition of EGF did not result in increased cell numbers. Removal of FGF2 from the basal culture medium also did not affect cell numbers. This however might be caused by a delayed effect due to FGF2 being present during the first 2 days in culture. Notch inhibition by DAPT also caused a modest reduction in pancreas progenitors and showed higher levels of *CHGA* and *NEUROG3*, along with the other notch inhibitor tested (GSiXX) (Fig. 6b, Supplementary Fig. 5a). The most pronounced effect in cell number increase was caused by activation of the WNT pathway, both by treatment with the GSK-3 inhibitor CHIR99021 or supplementation with WNT3a and R-Spondin. A modest effect on cell numbers was also observed upon both activation and inactivation of the RA pathway, inhibition of the BMP pathway via LDN193189, or treatment with PGE2.

Measuring the levels of ATP as a proxy for cell number (and implicitly proliferation) is suitable for screening but presents many confounding factors (e.g., metabolic changes). Thus, we proceeded to validate the most promising conditions for changes in proliferation. The assay timing was identical to the initial screen with one exception: due to the drastic effect seen upon treatment with Infigratinib, we restricted treatment with this compound to 24 h (on day 9 after re-plating) (Fig. 6c). We then proceeded to measure the levels of EdU+ cells at days 5 (3 days treatment) and 10 (8 days treatment) (Fig. 6c). After 3 days of treatment, the only condition resulting in increased percentage of EdU+ cells by flow cytometry was treatment with WNT3a and R-Spondin (Fig. 6d). This was also confirmed by the increased size of organoids observed by immunocytochemistry at day 10 (Fig. 6e). However, at day 10 neither EdU flow analysis nor immunostaining showed a sustained increase in proliferation. We also noted that WNT3a + R-Spondin treatment globally decreased the levels of PDX1 (Fig. 6e). Complementarily, we verified that inhibition of the WNT pathway in basal conditions with the porcupine inhibitor IWP-L6 did not result in decreased proliferation. After 8 days of treatment with Gefitinib or FGF2 removal, a decreased percentage of EdU+ cells was observed (Fig. 6d). Treatment with Infigratinib also resulted in a drastic decrease in proliferating cells after just 24 h of treatment (Fig. 6d).

To further test the relevance of these results, we repeated the same assay validation on fetal spheres (Supplementary Fig. 5b). We observed similar results overall, with the exception being that no increase in proliferation was seen after treatment with WNT3a + R-spondin (Supplementary Fig. 5c).

**Fig. 7:**
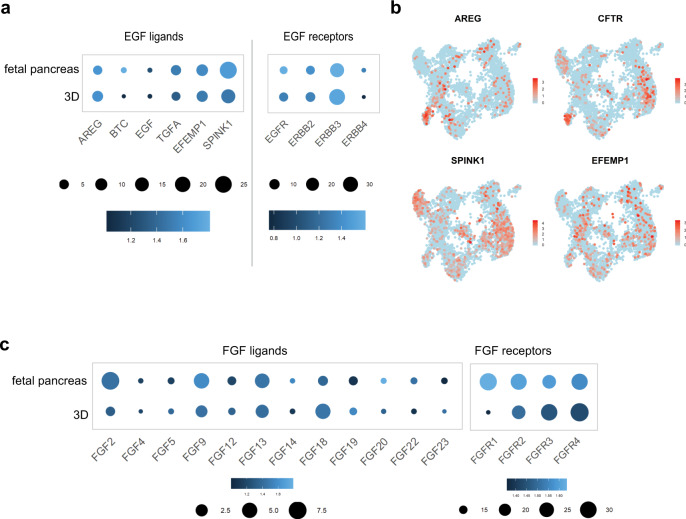
Expression of EGF and FGF pathways components. **a** Dot plot depicting expression of relevant EGF ligands and receptors in pooled 3D samples—PP-spheroids and fetal spheres—and fetal pancreas samples. **b** Expression of selected gene markers overlapped on the global UMAP showing *AREG* expressing cells enriched in the ductal cluster. **c** Dot plot depicting expression of FGF ligands and receptors in 3D samples—PP-spheroids and fetal spheres—and fetal pancreas. Color intensity indicates mean expression in a cluster, dot size indicates the proportion of cells in a cluster expressing the gene.

The effect of EGF signaling inhibition (Fig. 6) made us revisit the single-cell sequencing data to investigate the expression of pathway components. In addition to the EGF ligands *TGFA*, *EFEMP1*, and *SPINK1* revealed in vivo by InterCom (Fig. 1h), we detected a fourth possible ligand, *AREG* both in the fetal pancreas and in in vitro samples (Fig. 7a). Even though these cells represented a small proportion (4%) of the overall population, they clustered distinctly in the merged dataset (Figs. 3c, d and 7b). The same cells also formed a prominent cluster within the PP-spheroid and fetal spheres isolated datasets, but not in the 2D progenitors (Supplementary Fig. 3e, h). In the same cluster, we found higher levels of *CXCL8* and *FGF19*, both of which are involved in the *AREG* signaling cascade^54,55^ (Fig. 7b, d). SPINK1 was also enriched in the clusters expressing *CFTR*, while *EFEMP1* and *TGFA* were more homogeneously distributed (Fig. 7b). In addition, both *EGFR* and *ERBB3* were expressed in the 3D samples, as well as *ERBB2* and *4* to a lower degree (Fig. 7a). There were overall very few cells expressing FGF ligands at detectable levels, but notably the levels of *FGF2* were significantly lower in 3D samples compared to human fetal pancreas, suggesting that pancreatic progenitors respond to the FGF2 present in the medium by downregulating endogenous expression (Fig. 7c).

## Discussion

In this study, we developed a system to investigate early human fetal pancreas development in three dimensions, focusing on maintaining pancreas progenitors in culture, in a state close to their in vivo counterparts. The robustness of the system makes it appropriate for high throughput screening with small molecules, or in the future CRISPR screening to investigate the mechanisms of human pancreas development such as progenitor maintenance and expansion, ductal network formation, or endocrine induction. Moreover, it would enable to test the effect of genes identified by human geneticists that predispose to diabetes or other pancreatic affections on pancreatic development. While most hESC-derived pancreas differentiation methods have been focused on the production of β cells for diabetes therapy, our system is different in its aims, attempting to trap progenitors in vitro in a state as close possible to in vivo rather than forcing their differentiation in non-physiological proportions and times. As a system to study human pancreas development, we thus put emphasis on comparing the cells to those we also characterize in vivo in a single-cell transcriptome atlas.

### Atlas of human pancreas development

Here we present a single-cell level transcriptomic dataset of the human fetal pancreas at the first trimester (7–10 wpc). This study expands our previous global transcriptome of sorted populations^9^ by providing a single-cell resolution and distinguishing different progenitor populations. We corroborated the expression of *SOX9*, *PDX1*, and *KRT19* in pancreas progenitors up until 10 weeks^5,11,56,57^. Tip and trunk progenitor populations have been well characterized in mice^58^ and also suggested in human, marked by the loss of GATA4 in the central duct-like structures of the organ^57^ We further characterized the different progenitor populations, by comparing multiple genes differentially expressed between tip/trunk in mouse and observed the same behavior in the human fetal pancreas. We identified a population of tip progenitors, with higher expression of *GATA4, PTF1A, NR5A2*, and *CEL*, among others compared to the trunk clusters (Fig. 1f). Within this population, a small number of cells expressed *PRSS1* but lacked further markers of maturity. This is in accordance with other reports where onset of acinar specification was seen by 7 wpc^57,59^. In addition to the genes mentioned above, we identified others associated with this tip progenitor population (Supplementary Data 1). Similarly, we identified a population reminiscent of the mouse trunk progenitor population with increased levels of *SOX9*, *HNF1B*, *NKX6-1*, and *HES1* (Fig. 1e). Interestingly, the marker *SPP1* was also increased in trunk progenitors, although it is later restricted to the ductal epithelium in the adult pancreas^30^. *SPP1* has been shown to be expressed in progenitors during mouse development, and in light of our data the same may be true in human^60^. Our dataset corroborates previous observations on the time of endocrine commitment, with the first endocrine-committed cells appearing in the 7 + 3 wpc old fetal pancreas sample (~52 days)^5,57^. Further studies are warranted in order to test the putative progenitor maintenance function of the identified genes shown in Supplementary Data 1. Several genes whose mutations cause monogenic diabetes (*CEL* and *HNF1B*)^61–63^, predispose to type 2 diabetes *(RBPJL*)^64^, or cause cystic dysplasia (*NPHP3*)^65^ or exocrine insufficiency (*UBR1*)^66^ were detected in our dataset in these early stages of development. While we chose MARS-Seq as a technique that would allow for efficient capture of the scarce fetal samples and convenient collection of samples for optimal comparison (no batch effect), the number of cells remained in the thousands, possibly preventing us from identifying rare cell populations.

Comparison with published single-cell datasets in mice^32,33^ identifies differences in transcription between the two species including the ratio in functional homologs (*CPA1/2*), and important changes in gene expression level (*SOX9* higher in mice, *EGR1* in human).

A systematic analysis of the cell–cell communication network between all cell populations revealed interactions such as PDGFA-PDGFRA from the epithelium to the mesenchyme and others expected from mouse studies notably via EGFR and FGFR. Among those, combining the comparison between mouse and human EPs, we detect a conservation of receptors but many apparent differences in ligands. Further studies on the role of SEMA3C-NRP2 would be of interest as well as on SLIT2-ROBO1. The function of SLIT2 has not been studied in the pancreas, but a role of ROBO1/2 in promoting pancreas progenitor maintenance^41^, endocrine cell sorting^42^, and SLIT3 in endocrine differentiation^41^ suggest multiple activities of this family in pancreas development. Our data suggest predominant signaling activities of mesenchymal SLIT3 to mesenchymal ROBO1 in human development. As ROBO2 prevents stromal activation in the adult mouse^43^, ROBO1 may have a similar role in human development. While the activity of these pathways is suggested computationally, we functionally validated the EGF and FGF pathways, as further discussed below.

### A 3D culture system brings pancreatic progenitors closer to a fetal-like state

In order to mimic organ development, we found that by culturing hPSC-derived pancreatic progenitors in a 3D niche with minimal cytokine supplementation, we were able to obtain progenitors that more closely resembled a fetal EP than their 2D counterparts. Although single-cell transcriptome of pancreatic cell populations derived in vitro from PSCs has been reported^22^, the comparison with in vivo populations is an important assessment of their similarity and is a unique strength of our approach. Progenitors within our 3D culture system appeared to acquire a more defined pancreatic fate, decreasing the expression of liver marker genes that were more prevalent in 2D conditions (*TTR*, *APOE*, *AFP*, *APOA1*, *APOA2*). This effect was even more pronounced in later passages, where the expression of *APOA*, *APOE*, and *AFP* was reduced compared to early passages (Supplementary Fig. 3a, c). This suggests that either the change from a 2D to a 3D environment or the propagation for long periods of time is the main impulse in the acquisition of a more strict pancreatic fate. Although co-expression of liver and pancreatic markers in single cells has not been previously reported in pancreatic progenitors produced in vitro from hPSCs, the liver genes described here are also detected in publicly available datasets including bulk sequencing^67^ and single-cell sequencing^68^ using different differentiation protocols. Several are also detected in the early human pancreatic bud at 5 weeks although lower than in the liver primordium^8^. The liver markers downregulated in our dataset were also abundantly present prior to expansion in Trott et al.^46^ and were not consistently downregulated upon 2D expansion (Supplementary Data 4), arguing that the effect is either unique to our culture medium or to 3D conditions. While the composition of the culture medium may drive the expression of several genes, comparison between fetal spheres and PP-spheroids grown in the same medium was only partially overlapping (Fig. 3b), and several genes were specifically enriched in each of these two populations (Supplementary Fig. 3a). Practically, a robust and easy system for pancreas progenitor expansion is convenient, as experiments on pancreas progenitors can be conducted fast without necessitating lengthy hPSC expansion and differentiation. The system we developed enables the long-term maintenance and study of pancreas progenitors, complementing a previous culture system using feeder layers in 2D^46^. In addition, it enables the study of processes occurring in 3D, such as luminogenesis or cell delamination. The ability to differentiate into both α and β cells attests to the presence of progenitors at all stages, as well as the expression of NKX6-1, a gene that is normally lost in mature ducts. However, not all cells differentiate when subjected to differentiation protocols, which suggests that either feedback signals between cells limit differentiation (as known for the Notch pathway) or that PP-spheroids also contain more mature ductal cells that have lost the differentiation potential. Supporting this hypothesis, we show the presence of cells with ductal signatures and some heterogeneity in the expanded objects, both morphologically as well as by marker expression, a subset of objects exhibiting NKX6-1, MUC1, or CFTR (Fig. 2f, Supplementary Fig. 3b). Even though pancreas progenitors could be observed at all culture stages based on their markers and ability to differentiate, the endocrine differentiation after expansion is less efficient than in the best current pancreatic differentiation methods from hESCs^15,16,18,19,69^, and it is currently not the method of choice for mass production of endocrine cells for therapeutic purposes. Although *PTF1A* was induced after applying an acinar differentiation protocol, the limited efficiency of this protocol prevents us from concluding whether the progenitors we expanded are multipotent or bipotent.

Given its similarity to the fetal pancreas and ability to expand and differentiate, we believe this 3D culture method will be a useful tool to investigate morphogenetic processes that cannot be fairly accessed in 2D. The similarity in proliferation rates between the human fetal pancreas samples and fetal spheres indicates that our culture system maintains the innate replication capacity of progenitors, making this an excellent system to dissect self-renewal mechanisms of pancreatic progenitors.

### High reproducibility shows potential for high throughput screening

Having the ability to generate large numbers of pancreatic progenitors opens the door to high throughput in vitro experiments that could address timely questions about human pancreatic biology. As a proof of concept, we show that the culture system is amenable to screening. The screening identified the importance of EGFR and FGFR receptor signaling in pancreas progenitor expansion, extending previous observations in mouse and in human^70,71^. With respect to the FGF pathway, although FGF7 and FGF10 signaling from the mesenchyme have been proposed to expand pancreas progenitors in mouse and human, we show that at the stages we studied, *FGF7* and *FGF10* are expressed at much lower levels than *FGF2*, *FGF9,* and *FGF13* whose activities in the pancreas would be interesting to study. The receptors *FGFR1/2/3/4* were all broadly expressed in cells of the epithelium and mesenchyme (Fig. 7). Higher levels of *EGR1*, which is a target of the FGF pathway^70^—as well as other pathways—raise the possibility of a stronger activity of the pathway in human. In the screen we conducted, we could not see any requirement for YAP signaling in human pancreas progenitor expansion in contrast to previous reports in mouse and zebrafish proliferation^7^. This may be due to a lesser requirement in human or to the inability to detect any effect in the culture conditions we use, notably in the presence of potent mitogens such as FGF2. Treatment with WNT3a + R-Spondin resulted in transiently increased proliferation. WNT3a + R-Spondin have previously been used to expand fetal human pancreas progenitors in vitro but are not essential in our system, and as a drawback we observed reduced levels of PDX1 expression. We and others have previously reported that WNT/β-catenin signaling represses PDX1 in the mouse pancreas^72,73^, and a similar mechanism could be at play here.

The screening experiment also offered insights into the particular 3D culture system developed here. Contrary to previously published pancreatic organoid and sphere culture protocols, this method does not rely on EGF supplementation. We confirmed that addition of EGF does not result in increased proliferation, despite EGFR activity being required. Based on the abundance of ligands in vivo and in vitro, *TGFA*, *EFEMP1*, *SPINK1*, and *AREG* are the most likely ligands in human pancreas development at this stage. Interestingly, *AGR2*, which is highly upregulated in 3D comparing to both 2D and fetal samples has been shown to stimulate *AREG* expression leading to proliferation in the context of pancreas regeneration during pancreatitis^74^. In mouse BTC and EGF are the two ligands that were shown to activate the EGF pathway^75^, but they were not detected in our single-cell sequencing suggesting their expression levels are very low. This may reflect a species difference or a stage difference.

Overall, the system we characterized in great detail should enable further studies of pancreatic progenitors in a 3D environment. Conceptually, it shows that pancreatic progenitors can be captured and maintained in vitro, similarly to what has been achieved many years ago when stabilizing in vitro mouse and human PSCs in a state that is normally transient in the body. Since then, many studies have shown how different media make hPSCs more or less homogeneous and how they deviate from their attractor state. In the same spirit, the system should be amenable to study how transcriptional networks maintain pancreas progenitors in an attractor basin.

## Methods

### Human pluripotent stem cell lines and culture conditions

The H9 and H1 hESC lines were obtained from WiCell. The SBAD3.1 and SBAD3.4 iPSC lines were obtained from the stemBANCC consortium. The HuES4 PDX1-GFP reporter hESC line was previously reported^19^. All cell lines were approved for use in this project by De Videnskabsetiske Komiteer, Region Hovedstadenunder number H-4-2013-057. The hPSC lines were maintained in different conditions depending on the differentiation protocol to be performed. In the case of the protocol based on Ameri et al.^45^, the cells were maintained in the DEF-CS 500 (Takara). In the case of the protocol based on Rezania et al.^16^ the cells were maintained in mTeSR1 medium (Stem Cell Technologies) on hESC-Qualified LDEV-free Matrigel (Corning). Daily medium changes were performed, and all cells were cultured at 37 °C and 5% CO_2_. Cells were tested for mycoplasma free status regularly. When reaching ~80% confluence, cells were dissociated with TrypLE (Thermo Fisher), counted with the automated cell counter NucleoCounter NC-200 (ChemoMetec), washed in culture medium, and re-plated at a density of 40,000 cells/cm^2^. For passaging, mTeSR1 medium was supplemented with 10 µM ROCK inhibitor Y-27632 (Sigma) for the initial 24 h.

### Human samples

Human fetal pancreas tissue was obtained from material available following elective termination of pregnancy during the 1st trimester at the Departments of Gynaecology at Copenhagen University Hospital (Rigshospitalet) and Hvidovre Hospital, Denmark. The regional ethics committee “De Videnskabsetiske Komiteer Region Hovedstaden” approved this study (permit number H-1-2012-007), and women gave their informed written and oral consent. None of the terminations were for reasons of the pathology of pregnancy or fetal abnormality. Fetal age was determined by scanning crown-rump length and by evaluation of foot length and is indicated as estimated weeks + days post conception^76^. The pancreas was isolated, and the majority of the mesenchyme was removed manually.

### In vitro generation of pancreatic progenitors from human pluripotent stem cells

We generated pancreatic progenitors with two distinct protocols. Human PSCs were grown for at least three passages before starting the differentiation procedure.

Protocol adapted from Ameri et al.^45^: hPSCs (HuES4) were passaged as usual and allowed to reach confluency. The medium was changed daily, and 1× PBS (Thermo Fisher) washes were performed when changing media between stages. Cells were cultured in 2D through all stages of the protocols. Stage 1 (1 day): cells were exposed to RPMI GlutaMAX medium (Thermo Fisher) supplemented with 100 ng/ml Activin A (Peprotech), 3 µM CHIR 99021 (Axon), and B-27 supplement without insulin (Thermo Fisher). Stage 2 (4 days): cells were exposed to RPMI GlutaMAX medium supplemented with 100 ng/ml Activin A and B-27 supplement without insulin. Stage 3 (3 days): cells were exposed to DMEM/F12 GlutaMAX medium (Thermo Fisher) supplemented with 2 µM Retinoic Acid (RA; Sigma) and B-27 supplement (Thermo Fisher). Stage 4 (8 days): cells were exposed to DMEM/F12 GlutaMAX medium supplemented with 64 ng/ml FGF2 (Peprotech) and B-27 supplement.

Protocol modified from Rezania et al.^16^: hPSCs (H9, H1, SBAD3.1 and SBAD3.4) were passaged into plates coated with growth factor reduced Matrigel (GFR-Matrigel; Corning) diluted 1/30 in DMEM/F12 GlutaMAX. Cells were seeded at 200.000–350.000 cells/cm^2^ in mTeSR1 with 10 µM ROCK inhibitor Y-27632 and incubated until confluent (24–48 h depending on the specific cell line). The medium was changed daily, and 1× PBS washes were performed when changing media with different supplementation. Cells were cultured in 2D through all stages of the protocol. Stage 1 (3 days): cells were exposed to MCDB 131 medium supplemented with 1.5 g/L sodium bicarbonate, 1× Glutamax (Thermo Fisher), 10 mM glucose (Fisher Scientific), and 0.5% fatty acid-free bovine serum albumin (BSA; Proliant). The medium was further supplemented with 3 µM CHIR 99021 and 100 ng/ml Activin A for the first 24 h, then 0.3 µM CHIR99021 and 100 ng/ml Activin A the following day, and 100 ng/ml Activin A for the final day. Stage 2 (2 days): cells were exposed to MCDB 131 medium supplemented with 1.5 g/L sodium bicarbonate, 1× Glutamax, 10 mM glucose, 0.5% BSA, 0.25 mM ascorbic acid (Sigma), and 50 ng/ml FGF7 (Peprotech). Stage 3 (2 days): cells were exposed to MCDB 131 medium supplemented with 2.5 g/L sodium bicarbonate, 1× Glutamax, 10 mM glucose, 2% BSA, 0.25 mM ascorbic acid, 1:200 ITS-X (Thermo Fisher), 50 ng/ml FGF7, 1 µm RA, 0.25 µM Sant-1 (Sigma), 100 nM LDN193189 (LDN; Stemgent), and 200 nM TPB (EMD Millipore). Stage 4 (3 days): cells were exposed to MCDB 131 medium supplemented with 2.5 g/L sodium bicarbonate, 1× Glutamax, 10 mM glucose, 2% BSA, 0.25 mM ascorbic acid, 1:200 ITS-X, 2 ng/ml FGF7, 0.1 µm retinoic acid, 0.25 µM Sant-1, 200 nM LDN, and 100 nM TPB.

### Generation and expansion of PP-spheroids

A step-by-step protocol describing the long-term 3-D PP-spheroid culture protocol can be found at Protocol Exchange^77^. Pancreatic progenitors generated from hPSCs (HuES4, H9, H1, SBAD3.1, and SBAD3.4) were dissociated at St4d8 of the Ameri protocol or St4d3 of the Rezania protocol using TrypLE Express Enzyme (Thermo Fisher) to generate a single cell suspension and ran through a 35 µm cell strainer. A sample was taken to access the efficiency of differentiation by FACS. Cells were considered for PP-spheroid formation if at least 60% of the cells were PDX1+. Prior to re-plating, the cells were washed with expansion medium (DMEM/F12 Glutamax, 10 µM ROCK inhibitor Y-27632, 64 ng/ml FGF2, and B-27 supplement) and resuspended in a mixture of three parts GFR-Matrigel and one part expansion medium. The cells were plated at a density of 1000 cells/µl of Matrigel mixture in Nunc cell culture treated four well dishes (Thermo Fisher). The Matrigel cell suspension was dispensed slowly in the center of pre-warmed plates in order to form a 3D dome, and medium was added after Matrigel polymerization. The medium was changed every 3 days until passaging. The presence of ROCK inhibitor Y-27632, while strictly required for passaging, was not required for all the cell lines throughout the entire culture time. However, in order to keep conditions as uniform as possible, we chose to keep ROCK inhibitor Y-27632 in the culture medium by default. After 10 days in culture, PP-spheroids were dissociated with either TrypLE or a dissociation solution containing 0.25% Trypsin (Thermo Fisher) and 5 mM EDTA (Thermo Fisher) in 1× PBS, for 5-8 min. The single cells were strained as mentioned previously, quantified with the NucleoCounter NC-200, and re-plated. Cells were frozen in a solution of 50% expansion medium, 40% fetal bovine serum (FBS; Sigma) or knockout serum replacement (Thermo Fisher), and 10% dimethyl sulfoxide (DMSO; Sigma).

### Karyogram of PP-spheroids

The karyogram was analyzed on metaphase spreads of H9 PP-spheroids at P5 (Organisation Genetische Diagnostik Institut für Klinische Genetik Medizinische Fakultät Carl Gustav Carus TU Dresden). In addition, H9 PP-spheroids at P12 were analyzed for copy number variants larger than 350,000 bp (Life&Brain GENOMICS, Bonn, Germany), and no larger chromosomal aberration was reported.

### Generation and expansion of fetal spheres

Following fetal pancreas dissection and mesenchyme removal, the sample was digested with 250 U/ml Collagenase Type IV (Thermo Fisher) for 10–20 min at 37 °C, combined with mechanical dissociation every 5–10 min by pipetting, until only small clumps of tissue remained. The enzyme was inactivated with FBS, and the cells were washed in expansion medium. Similar to the PP-spheroid preparation, cells were suspended in a mix of three parts GFR-Matrigel to one part expansion medium and plated in Nunc cell culture treated four well dishes. After 10–15 days, the fetal spheres were passaged by an identical procedure, except a shorter Collagenase Type IV incubation (5–10 min).

### Immunohistochemical analysis of PP-spheroids and fetal spheres

PP-spheroids and fetal spheres (from HuES4, H9 and H1) were fixed with 4% PFA (Thermo Fisher) for 20–30 min depending on size, washed three times in 1× PBS, and incubated in 15% sucrose solution (Sigma) until equilibrated (approximately 1 h). The tissue was then incubated in gelatin for 30 min at 40 °C, followed by incubation at 4 °C for 15 min. After solidified, the resulting blocks were frozen in isopentane (Sigma) between −60 and −70 °C for 1 min and immediately stored at −80 °C. PP-spheroids or fetal spheres were cryosectioned in 7-µm-thick slices using a CM1950 cryostat (Leica) and collected on Superfrost Plus Adhesion slides (Thermo Fisher). Slides were rehydrated in TBS-T—1× homemade tris-buffered saline (TBS) with 0.1% Triton X-100 (Sigma)—for 10 min and permeabilised in a solution of 0.25% Triton X-100 in 1× TBS for 10 min. The slides were then washed with TBS-T and incubated in CAS-Block (Thermo Fisher) for 1 h. Primary antibodies were added to CAS-Block at the recommended concentration and incubated overnight at 4 °C. After thoroughly washing out primary antibodies with TBS-T, secondary antibodies (Alexa Fluor conjugates) and DAPI (Thermo Fisher) were diluted in CAS-Block and applied to the slide for 1 h. The slides were again thoroughly washed with TBS-T and mounted with 50% glycerol (Sigma) in 1× PBS. Antibody sources and concentrations are indicated in Supplementary Table 1. All images were acquired using the Zeiss LSM 780 with Zeiss Zen Black software.

### Immunohistochemical analysis of fetal pancreas

Fetal pancreas samples were fixed with 4% PFA for 60 min, washed three times in 1× PBS, and incubated in 15% sucrose solution overnight. The remaining procedure was identical to the one described for Immunohistochemical analysis of PP-spheroids and fetal spheres.

### Whole-mount immunostainings of PP-spheroids and fetal spheres

PP-spheroids (from HuES4, H9, and H1) and fetal spheres were fixed with 4% PFA for 20–30 min depending on size, washed three times in 1× PBS, and then gradually dehydrated to methanol (Sigma), at which point they could be stored at −20 °C. When applicable, a quenching step was performed by incubating samples with 30% of a Methanol/DMSO/H_2_O_2_ mix (Sigma) at a ratio of 4/1/1 overnight in PBS. Following this, the sample was gradually rehydrated to 1× PBS and incubated in CAS-block overnight. Primary antibodies were added to CAS-Block at the recommended concentration and incubated 24–48 h at 4 °C. After thoroughly washing out primary antibodies with 1× PBS containing 0.1% Triton X-100 (PBS-T), secondary antibodies (Alexa Fluor conjugates) and DAPI or DRAQ5 (Thermo Fisher) were diluted in CAS-Block and incubated with the sample overnight at 4 °C. Samples were again thoroughly washed with PBS-T and mounted with 60% glycerol in 1× PBS. Antibody sources and concentrations are indicated in Supplementary Table 1. All images were taken using the Zeiss LSM 780 with Zen Black software or Leica TCS SP8 with LAS X software.

### Flow cytometry analysis of PP-spheroids

Dissociated cells (from HuES4, H9, H1 and SBAD3.4) were washed in 1× PBS, stained with an appropriate Ghost Dye (TONBO biosciences) in 1% BSA in PBS, washed in 1× PBS, and then fixed in 4% PFA for 20 min on ice. Fixed cells were washed in 1× PBS and then permeabilised in PBS with 5% donkey serum (Sigma) and 0.2% Triton X-100 for 30 min at 4 °C. Cells were incubated with primary antibodies in 1× PBS with 5% donkey serum and 0.1% Triton X100 overnight at 4 °C. The following day, cells were washed twice in 1× PBS and unconjugated antibodies were further incubated with secondary antibodies (Alexa Fluor conjugates) for 45 min. Antibody sources and concentrations are indicated in Supplementary Table 1. Cells were analyzed using an LSRFortessa or FACSAria III (BD Biosciences), and data were analyzed with the FACSDiva (BD Biosciences), FlowJo (BD Biosciences), and FCS Express 6 software (De Novo Software). Representative data analysis and gating strategy are exemplified in Supplementary Fig. 6.

### Reverse transcription—quantitative PCR analysis

For RNA purification, cells were washed in 1× PBS twice, lysed in RLT buffer (QIAGEN RNeasy Mini or Micro kit) containing 1% β-mercaptoethanol (Sigma) and stored at −80 °C until processing. Total RNA was isolated from PP-spheroids, fetal spheres, or fetal pancreas using the RNeasy kit according to the manufacturers’ instructions and digested with DNase I (QIAGEN) to remove genomic DNA. First-strand cDNA synthesis was performed with Superscript III system (Thermo FIsher) using random primers (Thermo Fisher) and amplified using SYBR-Green (Thermo Fisher). PCR primers were designed using Primer3Plus (Untergasser et al. 2012^78^) and validated for efficiency ranging between 95 and 105%. The primer sequences of the genes used in qRT-PCR are listed in Supplementary Table 2. For qPCR the StepOnePLUS Real-Time PCR System (Thermo Fisher) was used for 96-well plates or the LightCycler 480 II instrument (Roche) for 384 well plates. Expression values for each gene were normalized against ACTB and RPL7, using the delta CT or delta–delta CT methods.

### EdU detection in PP-spheroids and fetal spheres

PP-spheroids (HUES4, SBAD3.4) and fetal spheres were incubated with 10 µM EdU (Click-iT EdU kit; Thermo Fisher) in expansion medium for 4 h at 37 °C. Then, PP-spheroids were prepared for flow cytometry as described above, while fetal spheres were processed for whole-mount immunostaining. Permeabilisation, blocking, and Click-iT reaction for EdU detection were performed according to the manufacturer’s instructions. Representative data analysis and gating strategy for flow cytometry is exemplified in Supplementary Fig. 6.

### Endocrine and exocrine differentiation in PP-spheroids

After 10 days of culture, expansion medium was changed to induce endocrine or exocrine differentiation.

#### Endocrine differentiation (based on Stages 5 and 6 of the Rezania et al.^16^ protocol)

Stage 5 (3 days): PP-spheroids (from HuES4, H9, H1, and SBAD3.4) were exposed to MCDB 131 medium supplemented with 1.5 g/L sodium bicarbonate, 1× Glutamax, 20 mM glucose, 2% BSA, 1:200 ITS-X, 10 µM zinc sulfate (Sigma), 0.05 µm retinoic acid, 0.25 µM Sant-1, 100 nM LDN, 10 µM ALK5 inhibitor II (Enzo), 1 µM T3 (Sigma), and 10 µg/ml heparin (Sigma). The medium was changed daily. Stage 6 (7-14 days): PP-spheroids were exposed to MCDB 131 medium supplemented with 1.5 g/L sodium bicarbonate, 1× Glutamax, 20 mM glucose, 2% BSA, 1:200 ITS-X, 10 µM zinc sulfate, 100 nM LDN, 10 µM ALK5 inhibitor II, 1 µM T3, 10 µg/ml heparin, and 100 nM GSiXX (EMD Millipore). The medium was changed daily.

#### Exocrine differentiation (based on^52^)

Fifteen days treatment: PP-spheroids (H9) were exposed to DMEM/F12 GlutaMAX medium supplemented with 15 ng/ml FGF7, 10 mM nicotinamide (Sigma), 100 ng/ml Exendin4 (GLP1 analog, Sigma), B27, and N2 supplements (Thermo Fisher).

### Screening setup, ATP quantification, and validation

PP-spheroids (HUES4) at passage 3 were dissociated as described above. A Bravo liquid handling robot (Agilent) was used to deposit 8 µl of cell suspension in all but 3 wells of a 96-well OptiPlate (PerkinElmer). GFR-Matrigel mixture without cells was added to the last three wells, to serve as a negative control. For the first 48 h, PP-spheroids were cultured as normal, in an expansion medium. After 48 h expansion medium was removed, wells were washed with 1× PBS twice, and different media were added in triplicates, including 26 conditions and 3 controls (details on Supplementary Table 3). The medium was again changed at day 5 and 8. After a total of 10 days in culture, Cell Titer-Glo luciferase assay (Promega) was performed according to the manufacturer’s instructions, and luminosity was measured in the LUMIstar omega (BMG Labtech). The validation experiments were performed in Nunc 4 well plates with both the HUES4 and SBAD3.4 cell lines, with the same timeline used in the original screening experiment, except for Infigratinib, which was only added from day 9–10. Cells were collected at days 5 and 10 of culture for EdU quantification. Validation in fetal spheres was performed in 8-well µ-slides (Ibidi).

### Single-cell RNA-sequencing and analysis

Generation of single-cell transcriptomic profiling from PP-spheroids (HUES4, H1, and SBAD3.4), 2D cell cultures (HUES4), fetal spheres, and fetal pancreas was done using the MARS-seq protocol as described^25^. Single cells were sorted using either the BD FACSAria III (BD Biosciences) or the Sony SH800 (Sony). Successful library preparation was confirmed using the Fragment Analyser device (Agilent) with the DNA HS kit (Agilent). Libraries were sequenced using Illumina Next-seq 500. R1 and R2 fastq files were generated using bcl2fastq v2.19.1, and the pooling and well information from the sequence was extracted into a unique fastq file using umis v1.0.3. The reads were then filtered based on the pooling barcodes with 1 mismatch allowed. The poly-Ts at the end of the sequences were trimmed using cutadapt v1.18^79^. Reads were mapped to the human genome (GRCh38 together with ERCC92) using hisat2 v2.1.0^80^, bam files generated, sorted and indexed with samtools v1.7^81^, reads counted with featureCounts (subread v1.5.3)^82^ using Ensembl version 93, and the umis using umi_tools v1.0.0^83^. SCATER v1.12.2^84^ was used to exclude low quality cells based on three QC covariates (count depth, genes per cell, and fraction of mitochondrial genes). An average of 2500 reads per cell, and 1500 genes per cell was obtained with this method. Cells were assigned into G1, G2/M, or S phase using the cyclone function from Scran v1.12.1^47^. Further computational analysis was done using Seurat 3.0.3^85^. Top 2000 variable genes were calculated using the variance stabilizing transformation method, and data integration was performed using pre-computed anchorsets in order to eliminate sequencing batch effects. After running PCA on the scaled integrated data, uniform manifold approximation and projection (UMAP) dimensional reduction^86^ was run to visualize cells. Clusters of cells with similar expression patterns were determined by using the FindNeighbors() and FindClusters() functions in Seurat 3.0.3 (see scripts deposited on github for specific parameters). The FindAllMarkers() function in Seurat 3.0.3 was used in order to obtain lists of differentially expressed genes in defined populations across the dataset. Visualizations were generated using Seurat 3.0.3 and ggplot2^87^.

### Comparison of human fetal single-cell RNA sequencing data to mouse embryonic single-cell RNA sequencing data

Mouse embryonic scRNAseq data of the pancreas at stage E12.5 (E12.5 SW) was obtained from^32^ (https://www.ncbi.nlm.nih.gov/geo/query/acc.cgi?acc=GSM3140915). Mouse data was processed, and cluster identity was assigned with Seurat v3.1.4. To compare human fetal data to mouse data, human genes were assigned with mouse orthologs downloaded from Ensembl BioMart. To find matching orthologs, mouse genes had to match the criteria for “mouse orthology confidence = 1 (high)” and “mouse homology type = ortholog_one2one”. All human–mouse gene pairs were assigned with a new unique gene identifier. New unique identifiers were then used to replace the species-specific gene identifiers in the mouse and human (fetal data) Seurat objects. Human and mouse Seurat objects were then integrated with the functions FindIntegrationAnchors and IntegrateData in Seurat, followed by data scaling and clustering.

To compare gene expression between human and mouse clusters, the expression of the top 50 cluster markers from human clusters (tip, trunk, endocrine) where compared to the expressions in corresponding mouse clusters and visualized in a dot plot. If not stated otherwise, all steps were performed with R version 3.6.3.

### Cell–cell communication analysis

To enable the systematic analysis of cell–cell communication, we developed InterCom, an R-package for inferring functional receptor–ligand mediated cell–cell interactions by integrating extracellular binding events with intracellular signaling cascades and transcriptional regulation.

InterCom relies on a compendium of experimentally validated ligand–receptor interactions, intracellular signaling networks, and gene regulatory interactions. Experimentally validated ligand–receptor interactions have been collected from a previously published dataset^88^. Interactions were further curated by selecting only ligands being annotated as “Secreted” in Uniprot^89^. In addition, an intracellular signaling network scaffold was used from pathway interactions included in Omnipath^90^, Reactome^91^, and MetaCore from Thomson Reuters. In particular, solely interactions related to signal transmission, such as phosphorylation and ubiquitination events, have been collected from these databases. Finally, transcriptional regulatory interactions were obtained from MetaCore from Thomson Reuters for human genes. Only transcriptional regulatory interactions with known effects, i.e. activation or inhibition, were selected by filtering for direct interactions with reported effect being activation or inhibition.

The main workflow of InterCom consists of the following steps. First, for each cell type, we identified transcription factors whose expression is preserved across a subset of cells. In particular, we transformed the expression data into a binary format, in which TFs with at least one count become 1 while not-expressed TFs become −1, and select TFs expressed in the top five-percentile of cells.

Second, InterCom detects functional receptors inducing these preserved TFs. we employed a Markov Chain model of intracellular signaling, called SigHotSpotter, to identify high probability intermediate molecule^92^. In brief, SigHotSpotter uses single-cell RNA-seq data of a cell type and the initially assembled intracellular signaling network to create a state transition matrix representing the traversal of a signal through the network. The state transition matrix represents a finite discrete Markov Chain and is subsequently evolved to create the stationary distribution. The stationary distribution displays the intermediate molecules exhibiting the highest steady-state probabilities. These intermediate molecules are then connected to the first transcription factors in the signal transduction chain, and their transcriptional compatibility is assessed. Intermediate molecules are considered to be compatible, if the expression of the molecule and TF targets agree with respect to the effect of the signaling path in a significant number of cases (Hypergeometric test; *p* < 0.05). Similarly, receptors are connected to intermediate molecules, if there exists a path that is compatible. Eventually, only receptors that are co-expressed with their interface TFs and downstream targets in at least 5% of cells are retained.

Finally, ligand–receptor interactions are established between two cell types, if the receptor has a downstream effect, the ligand is expressed in more than 5% of cells, and interaction is present in the interaction scaffold. An interaction score is defined as the product of the average receptor expression and average ligand expression in all cells of a population expressing the receptor or ligand, respectively. Significance is assessed by comparing the scores of all potential cell-cell interactions contained in the scaffold between the two interacting cell types. Scores in the top decile are considered significant.

### Image analysis

Whole-mount images of fetal spheres were analyzed, and EdU quantification was obtained through semi-manual segmentation using the surface detection tool of the IMARIS software v9.0.2 (Bitplane). Quantification of MKI67 from histological samples of human fetal pancreas, fetal spheres, and PP-spheroids was performed using the ImageJ distribution Fiji (http://pacific.mpi-cbg.de/wiki/index.php/ Fiji). An automated script was used to perform the following steps: Gaussian blur (sigma = 3); set threshold (default dark); convert to mask; watershed; analyze particles (size = 6, show outlines); measure intensity in each region of interest (ROI). The signal intensity threshold was manually determined for each sample.

### Quantification and statistical analysis

Statistical tests were performed using GraphPad Prism (6–8). Significance was defined as *P < 0.05, **P < 0.01, ***P < 0.001, ****P < 0.0001. *P* values were determined by the two-sided Mann–Whitney test unless otherwise stated. The number of replicates is indicated in the figure legends, with *N* denoting the number of independent experiments and *n* denoting the overall number of measurements. Unless otherwise stated, data are represented as the mean ± standard error of mean (SEM).

### Reporting summary

Further information on research design is available in the Nature Research Reporting Summary linked to this article.

## Supplementary information

Supplementary Information

Supplementary Data 1

Supplementary Data 2

Supplementary Data 3

Supplementary Data 4

Reporting Summary

Description of Additional Supplementary Files

## Data Availability

The raw single-cell sequencing datasets have been deposited in the European Genome-Phenome Archive EGA (https://ega-archive.org/) under ID number EGAD00001007506 and processed data can be browsed in the UCSC cell browser (https://human-pancreas-dev.cells.ucsc.edu). Figures that have associated raw data are Figs. 1, 3, 7 and Supplementary Figs. 1, 3, 7. We used Ensembl^93^ for gene annotation and homologous gene identifications. For InterCom analyses multiple protein sequence databases were used including Uniprot^89^, Omnipath^90^, Reactome^91^, and MetaCore from Thomson Reuters. The authors declare that the data supporting the findings of this study are available in the paper and its supplementary information files, and all relevant data are available from the authors.

## Code availability

The InterCom source code is available at https://github.com/saschajung/InterCom. Other codes are available at https://dx.doi.org/21.11101/0000-0007-E7E3-4.
