# Supplementary material for: A 3D system to model human pancreas development and its reference single-cell transcriptome atlas identify signaling pathways required for progenitor expansion
Source: Nat Commun. 2021 May 25;12:3144. doi: 10.1038/s41467-021-23295-6 (PMC8149728; doi:10.1038/s41467-021-23295-6)
Supplement: Supplementary file 1 — Supplementary Information [file 41467_2021_23295_MOESM1_ESM.pdf]

SUPPLEMENTARY INFORMATION

A 3D system to model human pancreas development and its reference single cell transcriptome atlas identify signaling pathways required for progenitor expansion

Carla A. Gonçalves[1], Michael Larsen[1], Sascha Jung[2], Johannes Stratmann[3], Akiko Nakamura[1], Marit Leuschner[3], Lena Hersemann[3], Rashmiparvathi Keshara[3], Signe Perlman[4], Lene Lundvall[4], Lea Langhoff Thuesen[5], Kristine Juul Hare[5], Ido Amit[6], Anne Jørgensen[7], Yung Hae Kim[3], Antonio del Sol[2,8,9], Anne Grapin-Botton[1,3*]

[1] The Novo Nordisk Foundation Center for Stem Cell Biology, Blegdamsvej 3B 2200 Copenhagen, Denmark

[2] CIC bioGUNE-BRTA (Basque Research and Technology Alliance), Bizkaia Technology Park, 801 Building, 48160 Derio, Spain

[3] Max Planck Institute of Molecular Cell Biology and Genetics, Pfotenhauerstr. 108, 01307 Dresden, Germany

[4] Department of Gynaecology, University Hospital of Copenhagen (Rigshospitalet), Blegdamsvej 9, 2100 Copenhagen, Denmark

[5] Department of Obstetrics and Gynaecology, Hvidovre University Hospital, Kettegård Alle 30, 2650 Hvidovre, Denmark

[6] The Weizmann institute, 234 Herzl Street, POB 26, Rehovot 7610001, Israel

[7] Department of Growth and Reproduction, Copenhagen University Hospital (Righshospitalet), Blegdamsvej 9, 2100 Copenhagen, Denmark

[8] Luxembourg Centre for Systems Biomedicine (LCSB), University of Luxembourg, 6 Avenue du Swing, Esch-sur-Alzette, L-4367 Belvaux, Luxembourg

[9] IKERBASQUE, Basque Foundation for Science, 48013, Bilbao, Spain

[*] Lead contact, correspondence: botton@mpi-cbg.de

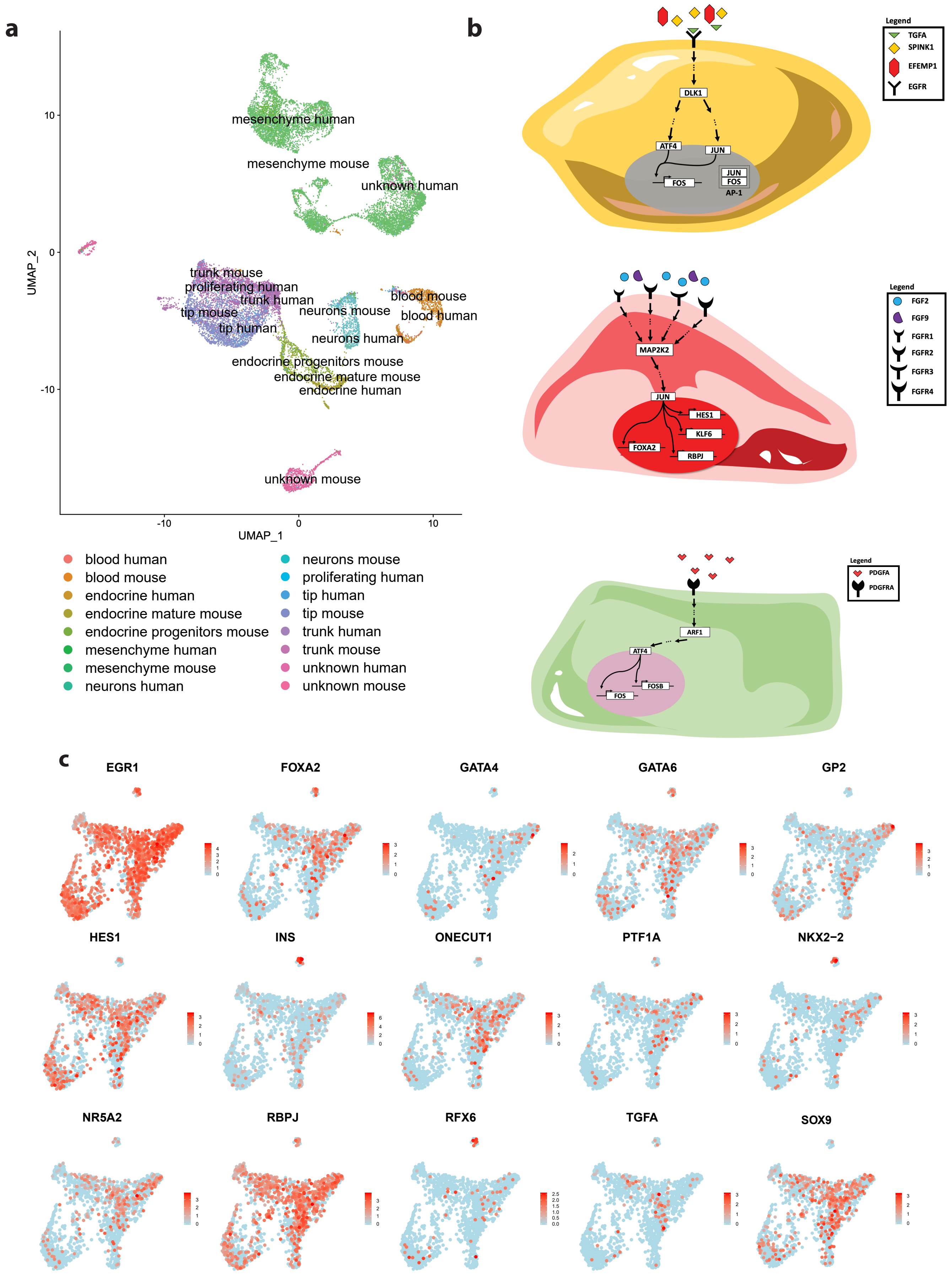

Supplementary Figure 1. Single cell transcriptome atlas of the fetal pancreas. Related to Figure 1. a UMAP plot representing human and mouse Seurat objects integrated with the function FindIntegrationAnchors and IntegrateData in Seurat, enabling to visualize matching populations in human and mouse. **b** Graphical representation of three example signaling pathways showing the ligands and receptors pairs and the intermediate signaling molecules with the highest likelihood and the transcription factors they are activating. **c** Distribution of known marker genes on UMAP.

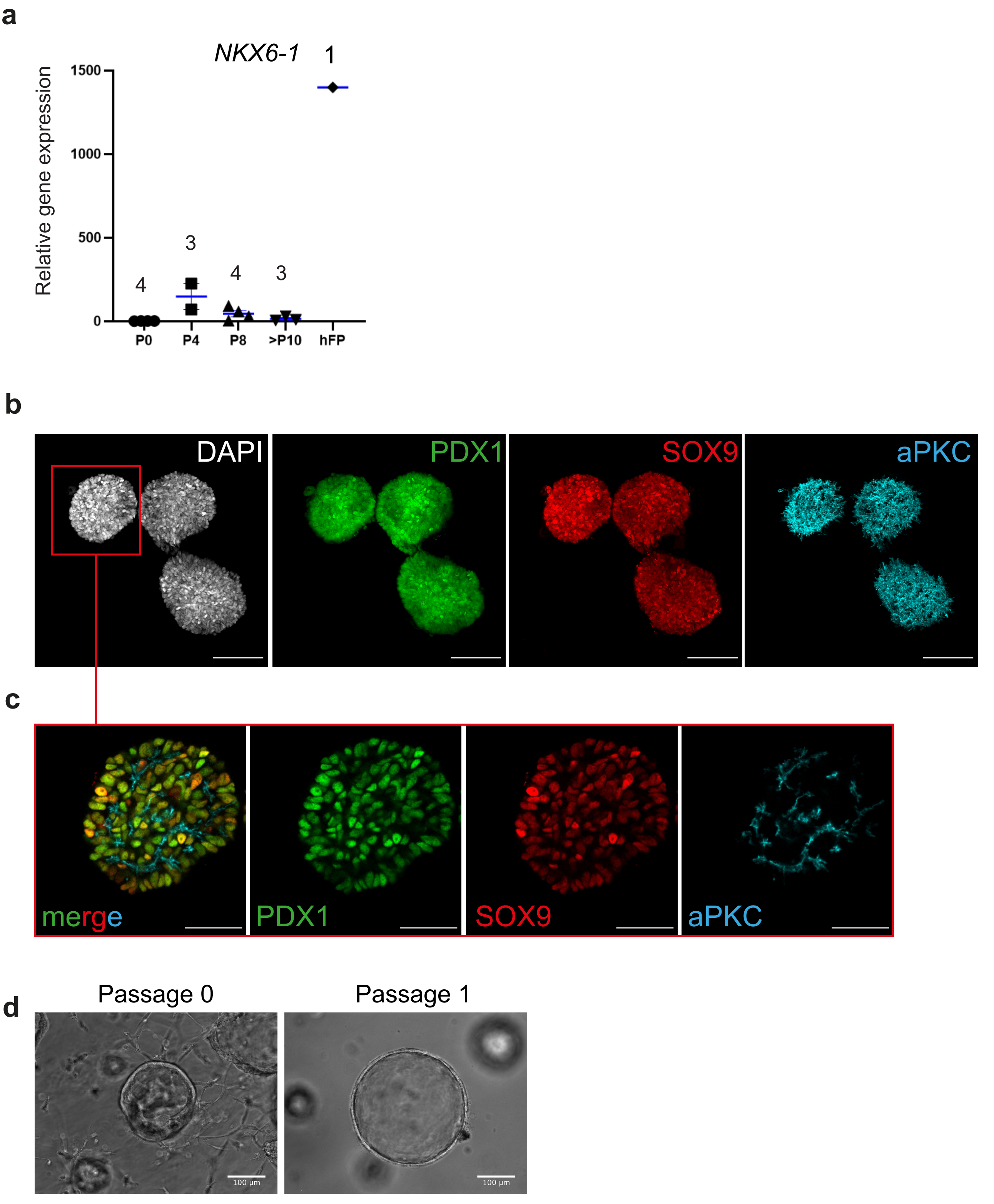

Supplementary Figure 2. A minimal culture system allowing long-term culture of human spheroids harboring pancreatic progenitors. Related to Figure 2. **a** qPCR analysis of *NKX6-1* shown in the samples where it was detected. Independent experiment number (N) is displayed above each column. A fetal pancreas sample (hFP, 7+3 wpc) was included as a control. Data shown as mean ± SEM. **b** Z-stack projection of whole-mount images of fetal spheres showing PDX1, SOX9, and aPKC, scale bar=50 µm. **c** Representative optical section of one of the fetal spheres, scale bar=25 µm. Images are representative of N=3 immunostainings. **d** Representative bright field images of fetal spheres show mesenchymal cells surrounding the epithelium at passage 0, and no mesenchymal cells present at passage 1, scale bar=100 µm. Images are representative of over N=4 experiments.

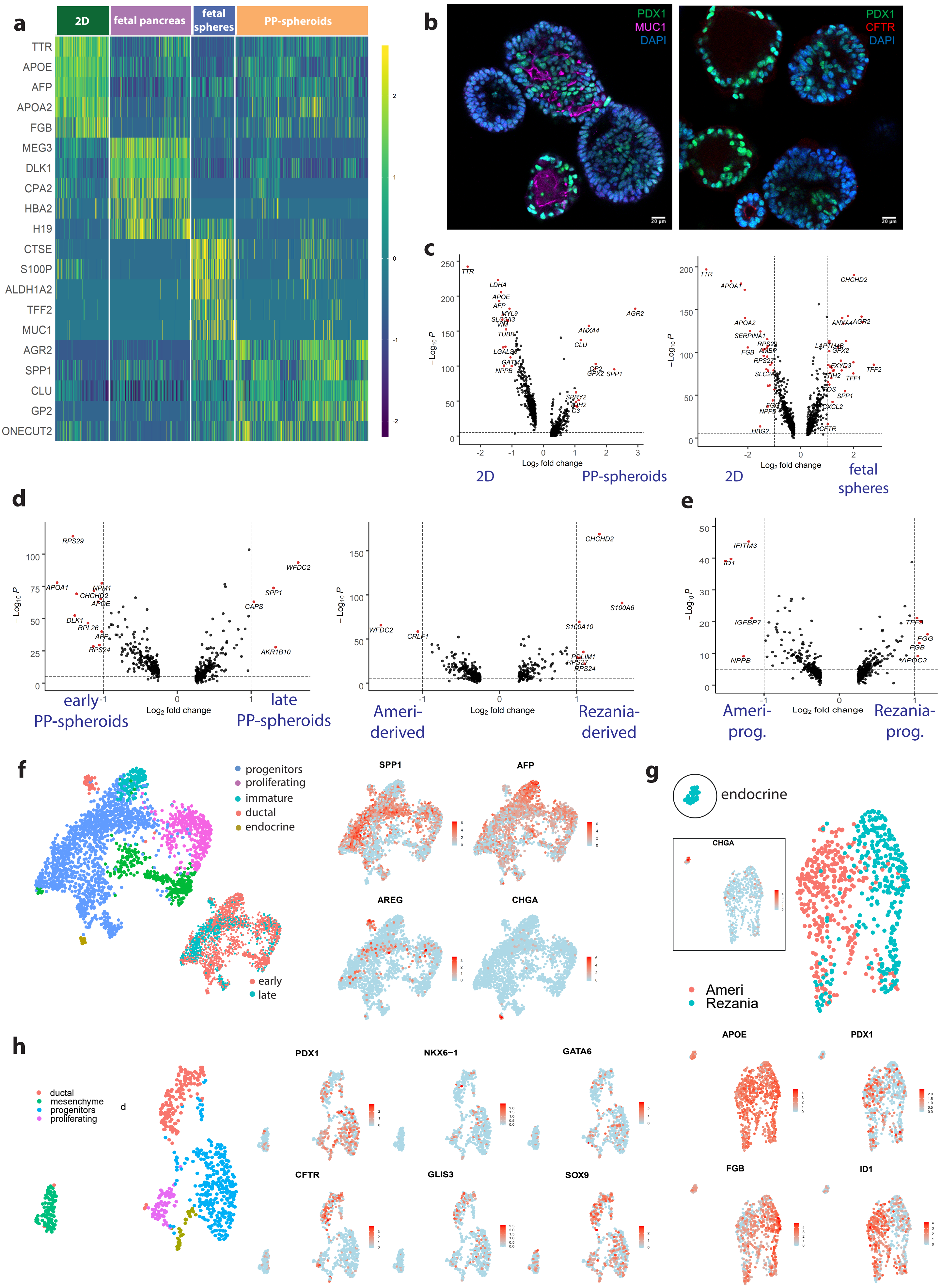
a
2D
fetal pancreas
fetal spheres
PP-spheroids
TTR
APOE
AFP
APOA2
FGB
MEG3
DLK1
CPA2
HBA2
H19
CTSE
S100P
ALDH1A2
TFF2
MUC1
AGR2
SPP1
CLU
GP2
ONECUT2
b
PDX1
MUC1
DAPI
PDX1
CFTR
DAPI
20 µm
c
Log2 fold change
2D
PP-spheroids
2D
fetal spheres
d
early PP-spheroids
late PP-spheroids
Ameri-derived
Rezania-derived
e
Ameri-prog.
Rezania-prog.
f
progenitors
proliferating
immature
ductal
endocrine
SPP1
AFP
AREG
CHGA
early
late
g
endocrine
CHGA
Ameri
Rezania
h
ductal
mesenchyme
progenitors
proliferating
PDX1
NKX6−1
GATA6
CFTR
GLIS3
SOX9
APOE
PDX1
FGB
ID1

Supplementary Figure 3. Transcriptome profile comparisons between samples and further annotations. Related to Figure 3. a A heatmap of the top 5 genes enriched in each sample. **b** Immunostaining of MUC1 and CFTR on day 10 PP-spheroids. Images are representative of N=2 experiments. **c** Volcano plots depicting the top differentially expressed genes between 2D progenitors and PP-spheroids or between 2D progenitors and fetal spheres. **d** Volcano plots depicting the top differentially expressed genes between early (passage 5) and late (passage 17) PP-spheroids; as well as the differentially expressed genes between PP-spheroids derived from the Ameri-based protocol and from the Rezania-based protocol. **e** PP-spheroids UMAP colored according to annotated clusters and early or late passage; UMAPs showing distribution of selected genes across the PP-spheroid population. **f** Volcano plot depicting the top differentially expressed genes between progenitors in 2D, differentiated with Ameri and Rezania protocols. Endocrine cells were excluded. **g** Differentiated progenitors in 2D UMAP colored according to the protocol used for differentiation; UMAP showing *CHGA* expressed in the endocrine cluster, and expression of *APOE* and *PDX1*. **h** Fetal spheres UMAP colored according to annotated clusters; UMAPs showing distribution of selected genes across the fetal spheres population. Non-annotated cell clusters (green in PP-spheroids and yellow in fetal spheres) were of uncertain identity.

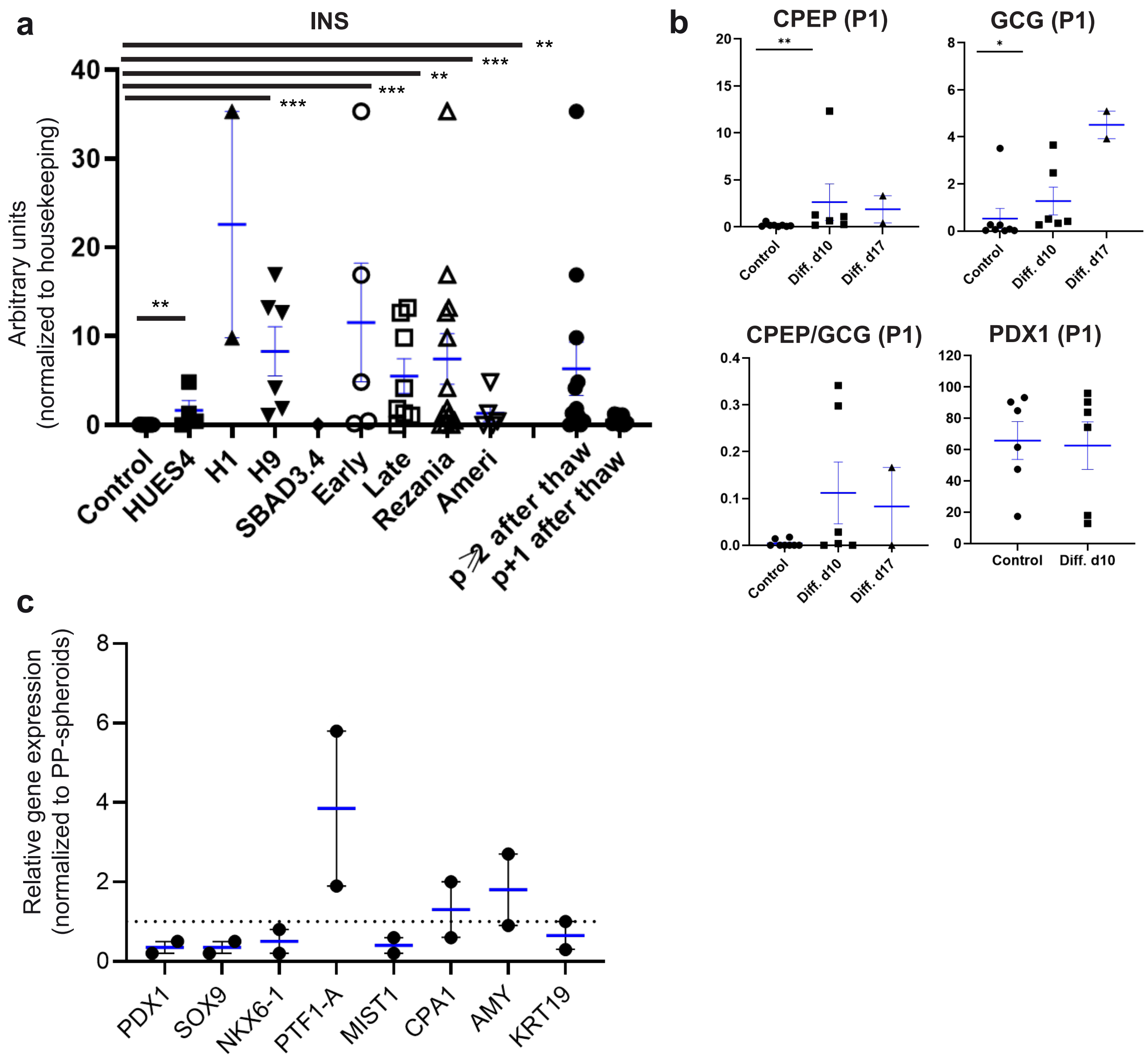

Supplementary Figure 4. Variable endocrine differentiation among cell lines and exocrine differentiation. Related to Figure 5. a qPCR analysis of INS split by cell line, passage time, pancreas progenitor differentiation protocol used and passage number after last thawing. Control N=16, HUES4 N=4, H1 N=2, H9 N=6, SBAD3.4 N=1, Early N=5, Late N=8, Rezania N=13, Ameri N=5, p≥2 after thaw N=4, p+1 after thaw N=8. **b** Flow cytometry data for INS, GCG and PDX1 showing the low differentiation capacity at passage 1 after the last thawing both at day 10 (N=6) and day 17 (N=2). This can be compared to the increased differentiation more than 1 passage after thawing shown in Fig. 5c. **c** Summary of qPCR analyses after exocrine differentiation. Expression levels were normalized to PP-spheroids, N=2. Data shown as mean ± SEM. *P < 0.05, **P < 0.01, ***P < 0.001. P values were determined by two-sided Mann-Whitney test.

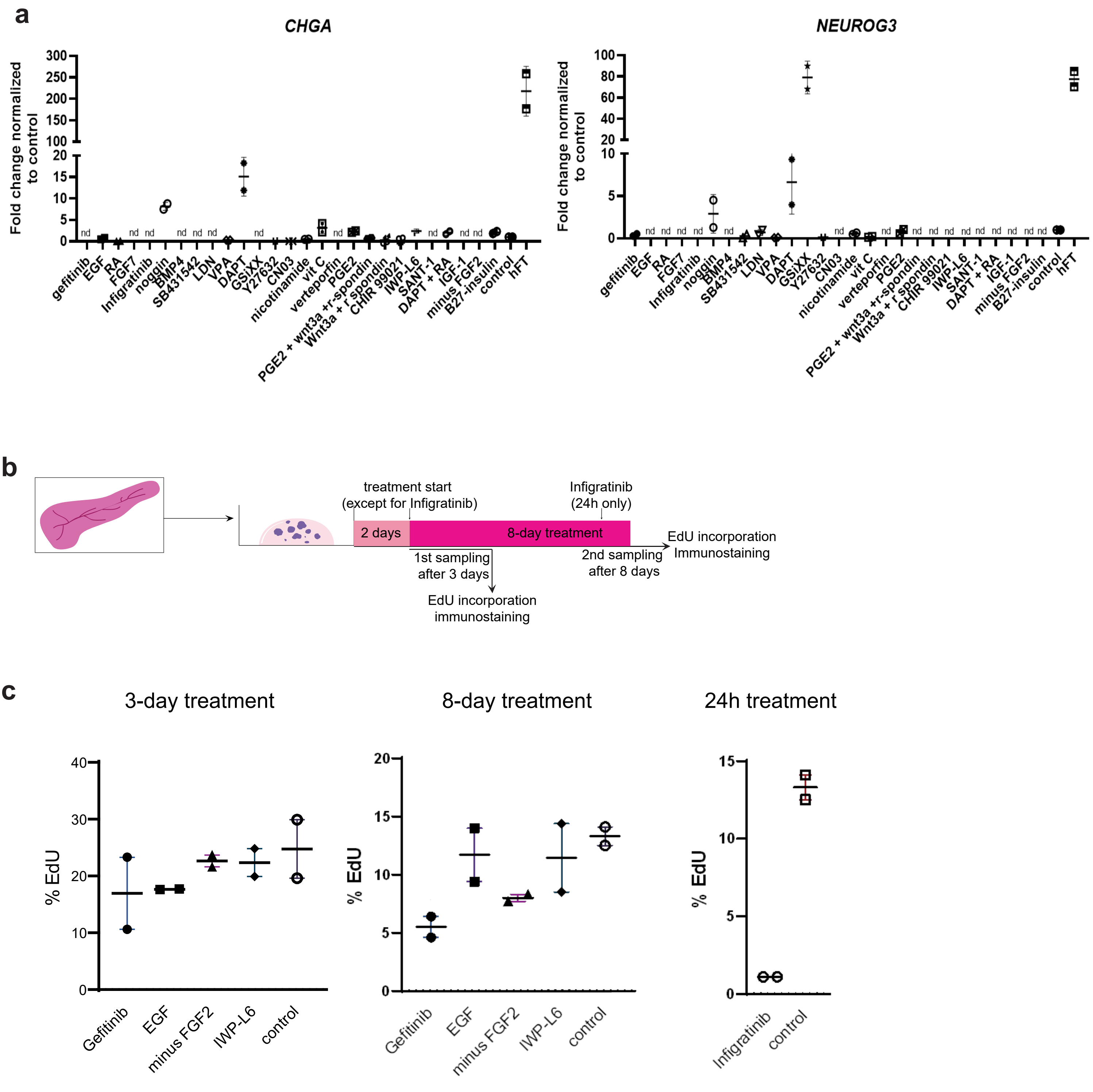

Supplementary Figure 5. Screening for endocrine differentiation in PP-spheroids and validation in fetal spheres. Related to Figure 6. a qPCR analysis of *CHGA* and *NEUROG3* for different conditions screened in PP-spheroids. Conditions where expression was not detected are omitted and labeled as nd. A fetal pancreas sample (9+3 wpc) was included as a control (hFT), n=2. Data shown as mean ± SD. **b** For screening validation, we performed EdU quantification by whole-mount immunostaining after 3 or 8 days of treatment. Treatment times were identical to the screening except for NVP-BGJ398 which was used for the last 24 hours only. **c** EdU percentages for different conditions after 3, 8 or 1 (for NVP-BGJ398) day of treatment, N=2. Data shown as mean ± SEM.

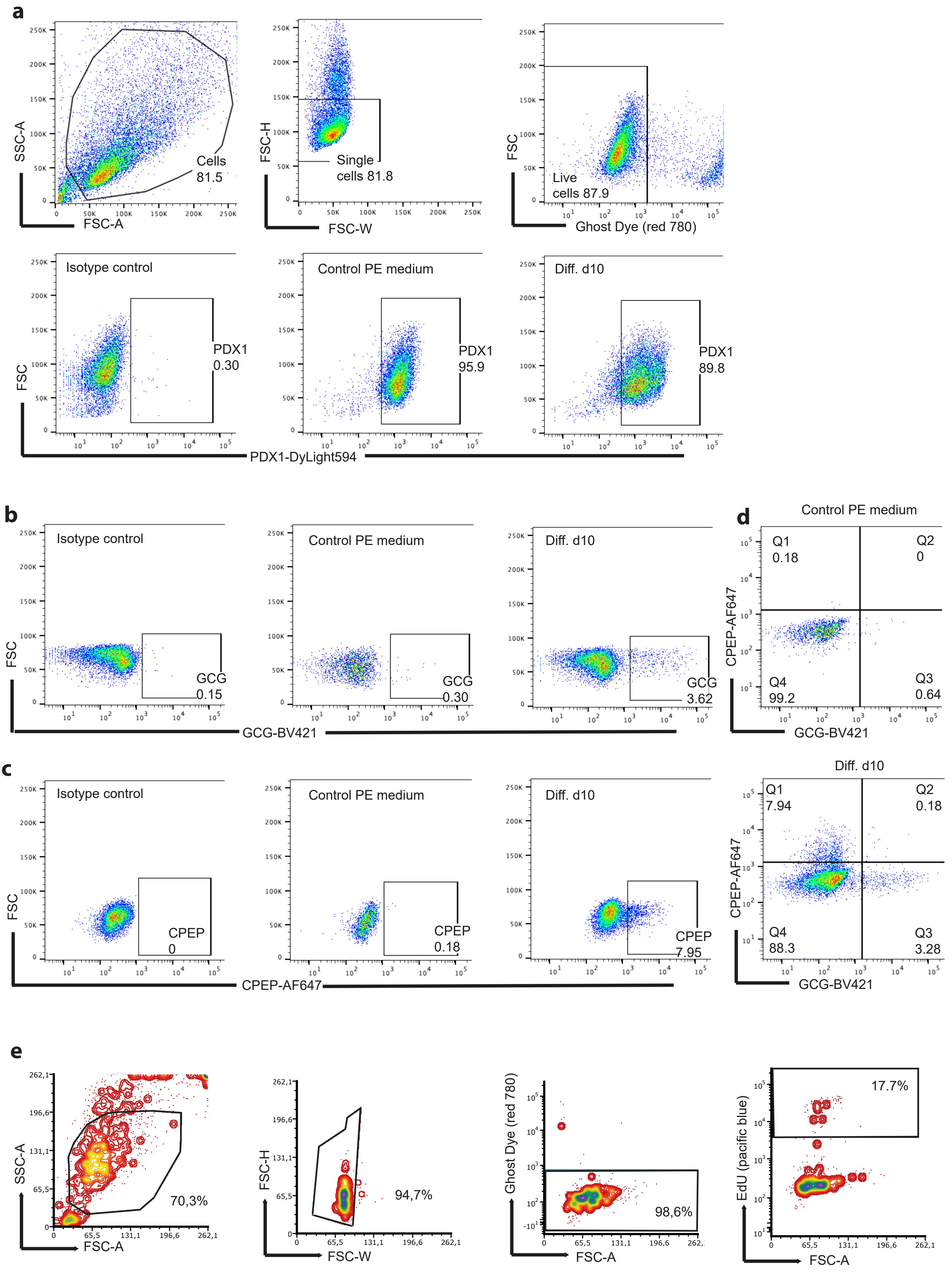
a
SSC-A
FSC-A
Cells 81.5
FSC-H
FSC-W
Single cells 81.8
FSC
Ghost Dye (red 780)
Live cells 87.9
Isotype control
PDX1 0.30
Control PE medium
PDX1 95.9
Diff. d10
PDX1 89.8
PDX1-DyLight594
b
Isotype control
GCG 0.15
Control PE medium
GCG 0.30
Diff. d10
GCG 3.62
GCG-BV421
d
Control PE medium
Q1 0.18
Q2 0
Q4 99.2
Q3 0.64
CPEP-AF647
c
Isotype control
CPEP 0
Control PE medium
CPEP 0.18
Diff. d10
CPEP 7.95
CPEP-AF647
Diff. d10
Q1 7.94
Q2 0.18
Q4 88.3
Q3 3.28
GCG-BV421
e
SSC-A
FSC-A
70,3%
FSC-H
FSC-W
94,7%
Ghost Dye (red 780)
98,6%
EdU (pacific blue)
17.7%

Supplementary Figure 6. Representative flow cytometry plots and gating strategy. a – d Representative flow cytometry pseudocolor plots related to Figure 5c and Supplementary Figure 4a, b. From a dissociated, stained cell population, single cells were selected, and then Ghost dye-negative cells were selected for analysis (a). From single live cells, positively stained cell populations were gated above the levels of fluorescence from antibody-specific isotype controls, as a negative control. Examples of positive populations of PDX1 (a), GCG (b), and C-PEPTIDE (c) are shown. From these data, double scatter plots are generated to determine double-positive cell populations (d). **e** Representative flow cytometry pseudocolor plots related to Figure 6d and Supplementary Figure 5c. From a single live cell population, EdU-positive population was gated based on fluorescent intensity.

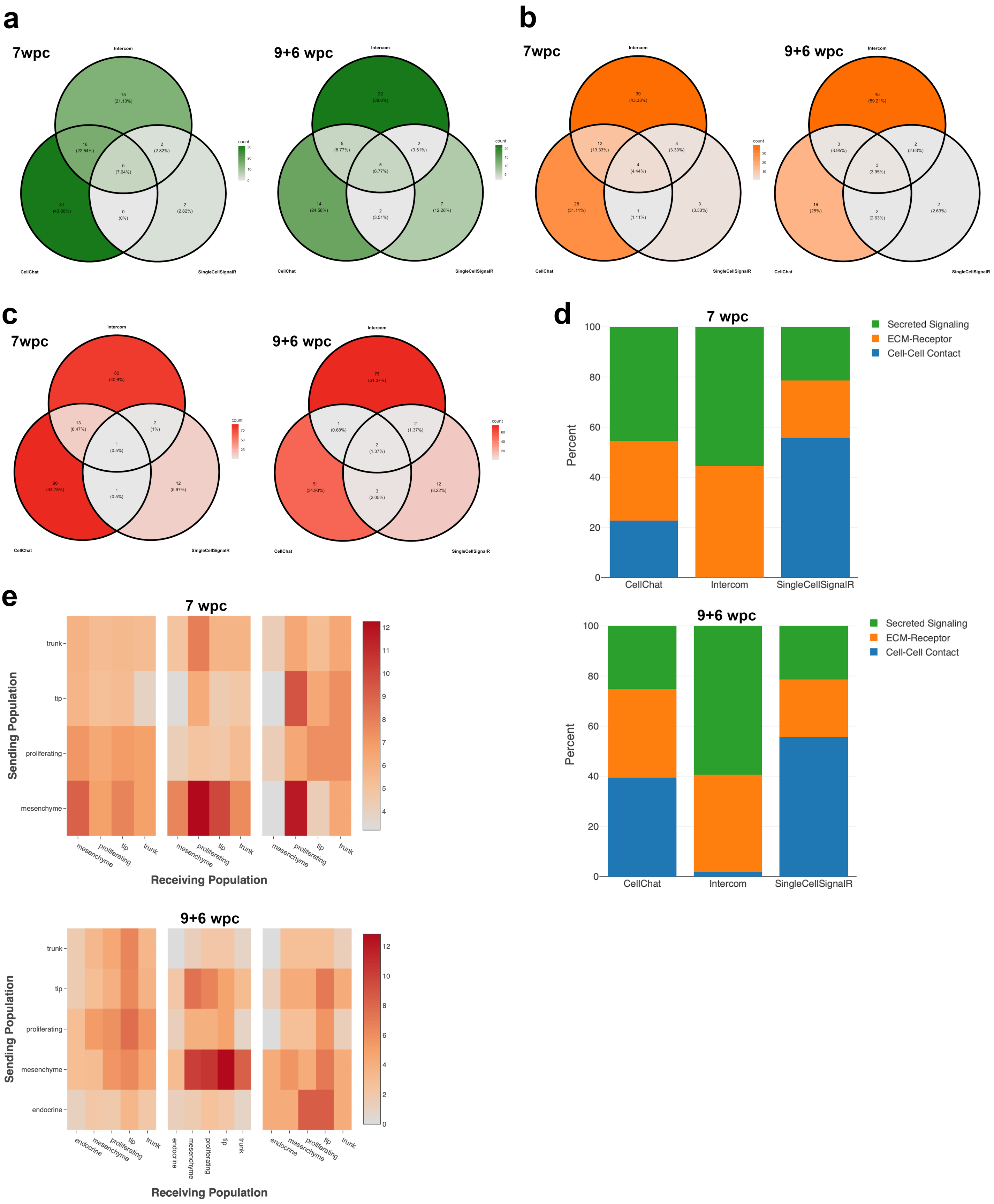

Supplementary Figure 7. Comparison of Intercom with current cell-cell communication network inference tools. a,b Overlap of unique (**a**) receptors and (**b**) ligands involved in the cell-cell interactions predicted by Intercom, CellChat and SingleCellSignalR for each sample (7 and 9+6 weeks post conception (wpc)). **c** Overlap of unique interactions in the communication networks reconstructed by Intercom, CellChat and SingleCellSignalR for each sample (7 and 9+6 wpc). **d** Frequency of interaction types in the reconstructed networks by Intercom, CellChat and SingleCellSignalR for each sample (7 and 9+6 wpc). **e** Heatmap depicting the frequency of interaction between different cell populations in the cell-cell communication networks inferred by each method. A gradient coloring represents the frequency from low (grey) to high (red).

Supplementary Table 1. Antibody sources and dilutions.

Antibodies	source	identifier	Dilutions
PDX1	R&D Systems	AF2419	1:500
SOX9	Millipore	AB5535	1:500
NKX6-1	DSHB	F55A10-c	1:100
EZRIN	Abcam	ab4069	1:500
CDH1	Sigma	U3254	1:200
aPKC	R&D Systems	AF259NA	1:500
MKI67	Abcam	ab16667	1:500
INSULIN	Dako	A0564	1:1000
C-peptide-Alexa Fluor 647 (flow cytometry)	BD Pharmingen	565831	1:200
Glucagon-BV421 (flow cytometry)	BD Pharmingen	565891	1:100
Ghost Dye Red 780	TONBO biosciences	13-0865	1:1000
Ghost Dye UV 450	TONBO biosciences	13-0868	1:1000
DAPI	Thermo Fisher	D1306	1 µM
DRAQ5	Cell Signaling Technology	4084	5 µM
Alexa fluor anti-goat 488	Abcam	ab150129	1:1000
Alexa fluor anti-goat 568	Thermo Fisher	A11057	1:1000
Alexa fluor andti-rabbit 568	Thermo Fisher	A10042	1:500
Alexa fluor anti-rabbit 647	Jackson Immuno Research Europe Ltd	711-605-152	1:1000
Alexa fluor anti-mouse 568	Thermo Fisher	A10037	1:1000
Alexa fluor anti-mouse 647	Jackson Immuno Research Europe Ltd	715-605-150	1:1000
Alexa fluor anti-rat 647	Jackson Immuno Research Europe Ltd	712-605-153	1:500
Alexa fluor anti-guinea pig 568	Thermo Fisher	A11075	1:1000
Goat IgG Isotype control	R&D Systems	AB-108-C	1:250
Mouse IgG1, k Isotype control-AF647	BD Pharmingen	557783	1:100
Mouse IgG1, k Isotype control-BV421	BD Pharmingen	562438	1:80

Supplementary Table 2. Sequence of primers used for qPCR.

Gene	Froward primer (5' to 3')	Reverse primer (5' to 3')
ACTB	CTC TTC CAG CCT TCC TTC CT	TGT TGG CGT ACA GGT CTT TG
AMY	GAGTAATGTCAAGCTACCGTTGG	TGTTCACAGACCCAGTCATTG
ARX	GCCATGAGGCTGGACTTGAC	GGCCCGACGGTTCTGGAA
CHGA	CTCCCTGTGAACAGCCCTA	TGTGTCGGAGATGACCTCAA
CPA	TTTCCAAGGCTGCTGTGAC	AGGTCCAGTCAATAGTGCTTCC
FOXA2	GGG AGC GGT GAA GAT GGA	TCA TGT TGC TCA CGG AGG AGT A
GCG	AAG CAT TTA CTT TGT GGC TGG ATT	TGA TCT GGA TTT CTC CTC TGT GTC T
GP2	GCACTCCCTGGTGTAATCTGA	CTGAGGCTGCAAACTGTGGA
HES1	AGC ACA CTT GGG TCT GTG C	TGA AGA AAG ATA GCT CGC GG
HNF1B	AGA GGT CCC TGC TTA CCT GAC	GCC TCC TGA GAG TGG ATT GT
INS	AAG AGG CCA TCA AGC AGA TCA	CAG GAG GCG CAT CCA CA
KRT19	CCACTACTACACGACCATCCA	AGGACAATCCTGGAGTTCTCAA
MAFA	AGAGCGAGAAGTGCCAACTC	GCCAGCTTCTCGTATTTCTCC
MIST1	CGGATGCACAAGCTAAATAACG	CCGTCAGCGATTTGATGTAGTTC
NEUROG3	GCT CAT CGC TCT CTA TTC TTT TGC	TCT CAC GGG TCA CTT GGA C
NKX6-1	CTG GCC TGT ACC CCT CAT CA	CTT CCC GTC TTT GTC CAA CAA
ONECUT1	CGC TCC GCT TAG CAG CAT	GTG TTG CCT CTA TCC TTC CCA T
PAX6	TTGCCCGAGAAAGACTAGCA	TCTCCATTTGGCCCTTCGATTA
PDX1	CCT TTC CCA TGG ATG AAG TC	GGA ACT CCT TCT CCA GCT CT
PTF1-A	AGAGAGTGTCCTGCTAGGGG	CCAGAAGGTCATCATCTGCC
RPL7	CAA AAG ATG CTT CGA AAG GC	TTT GGG TTC TGC AGG TAC ATA G
SOX9	GTA CCC GCA CTT GCA CAA C	TCT CGC TCT CGT TCA GAA GTC

Supplementary Table 3. Compounds used for screening assay and concentrations used.

Treatment	Final concentration
gefitinib	7 µM
EGF	50 ng/ml
BMS 493	100 nM
RA	3 µM
FGF7	50 ng/ml
Infigratinib	120 nM
noggin	100 ng/ml
BMP4	50 ng/ml
SB431542	10 µM
LDN	200 nM
VPA	10 µM
DAPT	1 µM
GSiXX	100 nM
nicotinamide	10 mM
vit C	250 µM
verteporfin	100 nM
PGE2	2.5 µM
PGE2 + wnt3a +r-spondin	2.5 µM + 100ng/mL + 500 ng/ml
Wnt3a + r spondin	100 ng/mL + 500 ng/ml
Chiron	3 µM
IWP-L6	5 µM
SANT-1	250 nM
DAPT + RA	13 µM +1 µM
human recombinant IGF-1	100 ng/ml
no FGF2	no FGF2 added after day 2
B27-insulin	B27 without insulin added instead of B27
control	sphere medium
DMSO control	0.1% DMSO in sphere medium
4 mM HCL control	20 µM HCL in sphere medium
empty	no cells

Supplementary Note 1. Comparison of Intercom with current Cell-Cell Communication Network Inference Methods

In order to demonstrate the advantages of Intercom, we set out to compare the cell-cell communication networks reconstructed by Intercom with those obtained by state-of-the-art tools. In particular, we employed three different tools, CellChat[1], SingleCellSignalR[2] and ICELLNET[3], which solely rely on a scRNA-seq dataset for network reconstruction. Although other tools, such as CellTalker[4] and iTALK[5], have been developed, we excluded them from this assessment as they only consider differentially expressed ligands and receptors.

To guarantee an unbiased analysis, most parameters have been set to their default values. However, we set the significance parameter of SingleCellSignalR to 0.9, which is the same cutoff employed in Intercom, and employed CellChat on projected data as recommended for sparse scRNA-seq samples. As a result, we were able to obtain reconstructed cell-cell communication networks from CellChat and SingleCellSignalR for the 7 and 9 wpc (weeks post conception) samples. However, ICELLNET failed to return networks of significant interactions even under varying parameter settings. Therefore, we excluded ICELLNET from the subsequent network comparison.

We first compared the reconstructed network quantitatively with respect to the number and type of interactions as well as the participating ligands and receptors. As a result, we observed vast differences between the networks reconstructed by Intercom, CellChat and SingleCellSignalR. In total, at most four ligands and five receptors participate in interactions inferred by all tools whereas up to 59% of molecules are unique to a single method (Supplementary Fig. 7a, b). As expected from these results, all methods predict vastly different interactions even without considering the sending and receiving cell populations (Supplementary Fig. 7c). To gain insight into the different types of interactions that are predicted by each method, we annotated each interaction as one of "Cell-Cell Contact", if it is an interaction between two membrane proteins, "ECM-Receptor", if the ligand is an extracellular matrix protein, and "Secreted Signaling", if the ligand is secreted and not an extracellular matrix protein. While CellChat provides this information as a result of the network reconstruction, we annotated the results of Intercom and SingleCellSignalR based on the Uniprot[6] location annotation of the ligand. In case it is annotated as "Secreted" and not as "Extracellular Matrix" the interaction is considered to be "Secreted Signaling". If the ligand is annotated as "Extracellular matrix" the interaction is considered to be "ECM-Receptor" interaction. Finally, in case both proteins are receptors that are not annotated to as "Secreted" the interaction is considered to be "Cell-Cell Contact". As a result, we observed that each method has a unique proportion of interaction types that is similar in both the 7 and 9 wpc networks (Supplementary Fig. 7d). For instance, while more than 50% of interactions in the SingleCellSignalR networks are related to cell-cell contact, the interaction types in the CellChat networks are more balanced. In contrast, between 50% and 60% of interactions in the Intercom networks belong to "Secreted Signaling" whereas the remaining interactions are "ECM-Receptor" interactions. However, we observed that 10 interactions in the 9+6 wpc network are classified as "Cell-Cell Contact" although only secreted ligands are included in the interaction scaffold. Nevertheless, these interactions all involve Ephrin A1 (EFNA1), a membrane protein that can be secreted as well. Finally, we assessed the interaction frequency between different cell populations in the networks

reconstructed by each method (Supplementary Fig. 7e). To account for the different number of interactions inferred by each method, we normalized the number interactions between a pair of cell populations by the total number of interactions. We observed that, depending on the interacting cell populations, the frequency of interactions in the Intercom networks is more similar either CellChat or SingleCellSignalR.

Despite the quantitative comparison, we qualitatively assessed the cell-cell communication networks of both CellChat and SingleCellSignalR with respect to the one identified by Intercom (Supplementary Data 3). For the 7 wpc, the communication network by SingleCellSignalR contained a total of 95 interactions out of which only six interactions matched with those of Intercom with respect to the ligand, the receptor as well as the sending and receiving cell populations. When examining the remaining 89 interactions, we found 27 false positives. More specifically, 25 of these interactions involve UBA52 as a ligand whereas the remaining two interactions are predicted to be between HSP90AA1 and FGFR3. However, UBA52 is neither secreted nor a membrane protein and HSP90AA1 is not a native ligand of FGFR3 (Supplementary Data 3). The remaining cell-cell interactions unique to SingleCellSignalR are related to Notch signaling (Notch1, Notch2, Notch3), Laminin signaling (RPSA) and ITGB1 signaling through midkine. Similarly, the 9 wpc communication network by SingleCellSignalR contains a total of 70 interactions out of which 8 interactions can also be found in the Intercom network. Overall, the interactions of the 7 and 9 wpc network are largely similar with 35 unique interactions related to Notch signaling (Supplementary dataset 1). Thus, the unique interactions predicted by SingleCellSignalR are largely related to cell-cell contact, which is expected given the design considerations of Intercom. However, the tool fails to predict important cell-cell interactions, such as Bmp and Fgf signaling.

Following the same rationale, we compared the cell-cell communication networks reconstructed by CellChat and Intercom. Since CellChat also contains receptor complexes, we considered an interaction to be common if Intercom predicts an interaction with at least one of their subunits. As a result, we observed that CellChat predicts 449 and 226 interactions for the 7 and 9 wpc sample, respectively, of which 10% and 3% are also predicted by Intercom (Supplementary Data 3). Similar to the SingleCellSignalR networks, the networks reconstructed by CellChat contain interactions related to cell-cell contact. In particular, CellChat predicts CD99, CDH1 and Notch signaling in both cases, which are not captured by Intercom. However, CellChat also predicts more than 45% of interactions whose participating ligand or receptor (subunit) is only expressed in few cells (below 5%). Notably, in some cases the ligand or receptor is not expressed in any cell (Supplementary Data 3). We speculate that this is due to CellChat's data transformation strategy to cope with sparse scRNA-seq data. The remaining interactions unique to the CellChat networks are related to NCL, NTRK2, Plexin and syndecan-2 signaling.

In summary, both CellChat and SingleCellSignalR are predicting many interactions that are unlikely to take place. Nevertheless, the cell-cell contact interactions these methods predict are complementary to the cell-cell communication network reconstructed by Intercom. Nevertheless, the absence of a ground truth network prevents a quantitative evaluation of the accuracy of each method. Especially CellChat potentially shows an improved performance when presented with a more dense expression dataset, for instance obtained from SmartSeq-based technologies.

Supplementary References

1. Jin, S. *et al.* Inference and analysis of cell-cell communication using CellChat. *Nat. Commun.* **12**, 1088 (2021).
2. Cabello-Aguilar, S. *et al.* SingleCellSignalR: inference of intercellular networks from single-cell transcriptomics. *Nucleic Acids Res.* **48**, e55–e55 (2020).
3. Noël, F. *et al.* Dissection of intercellular communication using the transcriptome-based framework ICELLNET. *Nat. Commun.* **12**, 1089 (2021).
4. Cillo, A. R. *et al.* Immune Landscape of Viral- and Carcinogen-Driven Head and Neck Cancer. *Immunity* **52**, 183-199.e9 (2020).
5. Wang, Y. *et al.* iTALK: an R Package to Characterize and Illustrate Intercellular Communication. *bioRxiv* 507871 (2019). doi:10.1101/507871
6. Bateman, A. *et al.* UniProt: the universal protein knowledgebase in 2021. *Nucleic Acids Res.* **49**, D480–D489 (2021).
