# Supplementary material for: A 3D system to model human pancreas development and its reference single-cell transcriptome atlas identify signaling pathways required for progenitor expansion
Source: Nat Commun. 2021 May 25;12:3144. doi: 10.1038/s41467-021-23295-6 (PMC8149728; doi:10.1038/s41467-021-23295-6)
Supplement: Supplementary file 6 — Reporting Summary [file 41467_2021_23295_MOESM6_ESM.pdf]

nature research

Corresponding author(s): Anne Grapin-Botton

Last updated by author(s): 2021-3-27

Reporting Summary

Nature Research wishes to improve the reproducibility of the work that we publish. This form provides structure for consistency and transparency in reporting. For further information on Nature Research policies, see our Editorial Policies and the Editorial Policy Checklist.

Statistics

For all statistical analyses, confirm that the following items are present in the figure legend, table legend, main text, or Methods section.

n/a	Confirmed	
☐	☒	The exact sample size (*n*) for each experimental group/condition, given as a discrete number and unit of measurement
☐	☒	A statement on whether measurements were taken from distinct samples or whether the same sample was measured repeatedly
☐	☒	The statistical test(s) used AND whether they are one- or two-sided<br>*Only common tests should be described solely by name; describe more complex techniques in the Methods section.*
☒	☐	A description of all covariates tested
☐	☒	A description of any assumptions or corrections, such as tests of normality and adjustment for multiple comparisons
☐	☒	A full description of the statistical parameters including central tendency (e.g. means) or other basic estimates (e.g. regression coefficient) AND variation (e.g. standard deviation) or associated estimates of uncertainty (e.g. confidence intervals)
☐	☒	For null hypothesis testing, the test statistic (e.g. *F*, *t*, *r*) with confidence intervals, effect sizes, degrees of freedom and *P* value noted<br>*Give P values as exact values whenever suitable.*
☒	☐	For Bayesian analysis, information on the choice of priors and Markov chain Monte Carlo settings
☒	☐	For hierarchical and complex designs, identification of the appropriate level for tests and full reporting of outcomes
☒	☐	Estimates of effect sizes (e.g. Cohen's *d*, Pearson's *r*), indicating how they were calculated

Our web collection on statistics for biologists contains articles on many of the points above.

Software and code

Policy information about availability of computer code

Data collection	Zeiss Zen Black software v14.0.22.201 and Leica LAS X v3.5.5.19976 were used to acquire images.<br>FACSDiva v8.0.1 (BD Biosciences) and Sony SH800 v1.8 were used to acquire FACS and flow cytometry data.<br>Softwares on StepOnePLUS Real-Time PCR System (Thermo Fisher) and the LightCycler 480 II instrument (Roche) were used to acquire and analyse PCR data.
Data analysis	After single cell sequencing, R1 and R2 fastq files were generated using bcl2fastq v2.19.1, the pooling and well information from the sequence was extracted into a unique fastq file using umis v1.0.3. The reads were then filtered based on the pooling barcodes with 1 mismatch allowed. The poly-Ts at the end of the sequences were trimmed using cutadapt v1.18 (Martin). Reads were mapped to the human genome (GRCh38 together with ERCC92) using hisat2 v2.1.0 (Kim et al., 2015), bam files generated, sorted and indexed with samtools v1.7 (Li et al., 2009), reads counted with featureCounts (subread v1.5.3) (Liao et al., 2014) using Ensembl version 93, and the umis using umi_tools v1.0.0 (Smith et al., 2017). SCATER v1.12.2 (McCarthy et al., 2017) was used to exclude low quality cells based on three QC covariates (count depth, genes per cell, and fraction of mitochondrial genes). An average of 2500 reads per cell, and 1500 genes per cell was obtained with this method. Cells were assigned into G1, G2/M or S phase using the cyclone function from Scran v1.12.1 (Scialdone et al., 2015). Further computational analysis was done using Seurat 3.0.3 (Stuart et al., 2019). Top 2000 variable genes were calculated using the variance stabilizing transformation (vst) method and data integration was performed using pre-computed anchorsets in order to eliminate sequencing batch effects. After running PCA on the scaled integrated data, uniform manifd0 approximation and projection (UMAP) dimensional reduction (McInnes et al., 2018) was run to visualize cells. Clusters of cells with similar expression patterns were determined by using the FindNeighbors() and FindClusters() functions in Seurat 3.0.3 (see scripts deposited on github for specific parameters). The FindAllMarkers() function in Seurat 3.0.3 was used in order to obtain lists of differentially expressed genes in defined populations across the dataset. Visualizations were generated using Seurat 3.0.3 and ggplot2 (Wickham, 2016). For mouse-human comparisons, Human and mouse Seurat objects were then integrated with the functions FindIntegrationAnchors and IntegrateData in Seurat, followed by data scaling and clustering.<br>To compare gene expression between human and mouse clusters, the expression of the top 50 cluster markers from human clusters (tip, trunk, endocrine) where compared to the expressions in corresponding mouse clusters and visualized in a dot plot. If not stated otherwise, all

steps were performed with R version 3.6.3.
Codes for single cell RNA sequencing data and comparison between mouse and human data are available at https://dx.doi.org/21.11101/0000-0007-E7E3-4.

For InterCom, we employed a Markov Chain model of intracellular signaling, called SigHotSpotter, to identify high probability intermediate molecule (Ravichandran et al., 2019).

The InterCom R package is available at https://github.com/saschajung/InterCom. Other codes are available at https://dx.doi.org/21.11101/0000-0007-E7E3-4

Whole mount images of fetal spheres were analyzed and EdU quantified through semi-manual segmentation using the surface detection tool of the IMARIS software v9.0.2 (Bitplane). Quantification of MKI67 from histological samples of human fetal pancreas, fetal spheres and PP-spheroids was performed using the ImageJ v1.52 distribution Fiji (http://pacific.mpi-cbg.de/wiki/index.php/ Fiji). An automated script was used to perform the following steps: Gaussian blur (sigma=3); set threshold (default dark); convert to mask; watershed; analyze particles (size=6, showoutlines); measure intensity in each region of interest (ROI). The signal intensity threshold was manually determined for each sample.

FACSDiva v8.0.1, FlowJo v10.7.1 (BD Biosciences), and FCS Express 6 software (De Novo Software) were used to analyse FACS and flow cytometry data.

Statistical tests were performed using GraphPad Prism (6-8).

For manuscripts utilizing custom algorithms or software that are central to the research but not yet described in published literature, software must be made available to editors and reviewers. We strongly encourage code deposition in a community repository (e.g. GitHub). See the Nature Research guidelines for submitting code & software for further information.

Data

Policy information about availability of data

All manuscripts must include a data availability statement. This statement should provide the following information, where applicable:

- Accession codes, unique identifiers, or web links for publicly available datasets
- A list of figures that have associated raw data
- A description of any restrictions on data availability

The raw single-cell sequencing datasets were deposited in EGA under ID number EGAD00001007506. We also deposited processed data in the UCSC cell browser (https://human-pancreas-dev.cells.ucsc.edu). Figures that have associated raw data are Figures 1, 3, 7 and Supplementary Figures 1, 3, 7. We used Ensembl (Flicek et al., 2010) for gene annotation and homologeous gene identifications. For InterCom analyses multiple protein sequence databases were used including Uniprot (UniProt, 2019), Omnipath (Turei et al., 2016), Reactome (Jassal et al., 2020), and MetaCore from Thomson Reuters. The authors declare that the data supporting the findings of this study are available in the paper and its supplementary information files.

Field-specific reporting

Please select the one below that is the best fit for your research. If you are not sure, read the appropriate sections before making your selection.

☒ Life sciences ☐ Behavioural & social sciences ☐ Ecological, evolutionary & environmental sciences

For a reference copy of the document with all sections, see nature.com/documents/nr-reporting-summary-flat.pdf

Life sciences study design

All studies must disclose on these points even when the disclosure is negative.

Sample size	Sample size was not predetermined by statistical methods. Sample size was set at a minimum of 3, and more samples were used when possible, though limited by tissue availability for experiments using human fetal pancreas. Sample numbers are indicated in the figure legends as N independent experiments containing n samples.
Data exclusions	From the single cell dataset, SCATER v1.12.2 (McCarthy et al., 2017) was used to exclude low quality cells based on three QC parameters (count depth, genes per cell, and fraction of mitochondrial genes). No other data was excluded.
Replication	All data were replicated in independent experiments (indicated as N in the figure legends) and no experiment was excluded.
Randomization	All treatment conditions during in vitro experiments were allocated randomly. The plate designs were chosen to minimize manipulation time. Blanks and functional controls were consistently included for experiments involving quantitative measurements in order to reduce the effect of confounders and covariates. For immunohistochemistry, the field of view was randomly selected for analysis.
Blinding	Due to the chemical nature of the compounds used for differentiation, drug screening, and validation experiments, blinding during treatment administration was not feasible. For molecular analyses, data collection and analysis arbitrary sample codes were used whenever possible, to minimize bias. Investigators were not blinded to group allocation during remaining in vitro data collection and analysis.

Reporting for specific materials, systems and methods

We require information from authors about some types of materials, experimental systems and methods used in many studies. Here, indicate whether each material, system or method listed is relevant to your study. If you are not sure if a list item applies to your research, read the appropriate section before selecting a response.

Materials & experimental systems

n/a	Involved in the study
☐	☒ Antibodies
☐	☒ Eukaryotic cell lines
☒	☐ Palaeontology and archaeology
☒	☐ Animals and other organisms
☐	☒ Human research participants
☒	☐ Clinical data
☒	☐ Dual use research of concern

Methods

n/a	Involved in the study
☒	☐ ChIP-seq
☐	☒ Flow cytometry
☒	☐ MRI-based neuroimaging

Antibodies

Antibodies used

PDX1 R&D Systems AF2419 1:500
SOX9 Millipore AB5535 1:500
NKX6-1 DSHB F55A10-c 1:100
EZRIN Abcam ab4069 1:500
CDH1 Sigma U3254 1:200
aPKC R&D Systems AF259NA 1:500
MKI67 Abcam ab16667 1:500
INSULIN Dako A0564 1:1000
C-peptide-Alexa Fluor 647 (flow cytometry) BD Pharmingen 565831 1:200
Glucagon-BV421 (flow cytometry) BD Pharmingen 565891 1:100
Alexa fluor anti-goat 488 Abcam ab150129 1:1000
Alexa fluor anti-goat 568 Thermo Fisher A11057 1:1000
Alexa fluor anti-rabbit 568 Thermo Fisher A10042 1:500
Alexa fluor anti-rabbit 647 Jackson Immuno Research Europe Ltd 711-605-152 1:1000
Alexa fluor anti-mouse 568 Thermo Fisher A10037 1:1000
Alexa fluor anti-mouse 647 Jackson Immuno Research Europe Ltd 715-605-150 1:1000
Alexa fluor anti-rat 647 Jackson Immuno Research Europe Ltd 712-605-153 1:500
Alexa fluor anti-guinea pig 568 Thermo Fisher A11075 1:1000
Goat IgG Isotype control R&D Systems AB-108-C 1:250
Mouse IgG1, k Isotype control-AF647 BD Pharmingen 557783 1:100
Mouse IgG1, k Isotype control-BV421 BD Pharmingen 562438 1:80

Validation

All antibodies have been validated by manufacturers at least by Western blot. They have also been reported in numerous other studies at least in mouse, not always in human, and we have ascertained their subcellular localization.

Eukaryotic cell lines

Policy information about cell lines

Cell line source(s)

human ES cell lines: HUES4 was obtained from Henrik Semb's lab, H9 and H1 from WiCell. SBAD3.1 and SBAD3.4 were were derived as part of the IMI-EU sponsored StemBANCC consortium and obtained from C. Honore.

Authentication

No cell line was authenticated.

Mycoplasma contamination

All cell lines tested negative for mycoplasma and were tested routinely.

Commonly misidentified lines (See ICLAC register)

None of the lines used are reported in ICLAC register.

Human research participants

Policy information about studies involving human research participants

Population characteristics	Human fetal pancreas tissue between 7 and 10 post-concenptional weeks of develpment was obtained from material available following elective termination of pregnancy during the 1st trimester at the Departments of Gynaecology at Copenhagen University Hospital (Rigshospitalet) and Hvidovre Hospital, Denmark. None of the terminations were for reasons of pathology of pregnancy or fetal abnormality.
Recruitment	See above. No selection bias.
Ethics oversight	The regional ethics committee (De Videnskabsetiske Komiteer Region Hovedstaden) approved this study (permit number H-1-2012-007) and women gave their informed written and oral consent.

Note that full information on the approval of the study protocol must also be provided in the manuscript.

Flow Cytometry

Plots

Confirm that:

- [x] The axis labels state the marker and fluorochrome used (e.g. CD4-FITC).
- [x] The axis scales are clearly visible. Include numbers along axes only for bottom left plot of group (a 'group' is an analysis of identical markers).
- [x] All plots are contour plots with outliers or pseudocolor plots.
- [x] A numerical value for number of cells or percentage (with statistics) is provided.

Methodology

Sample preparation	Dissociated cells (from HuES4, H9, H1 and SBAD3.4) were washed in 1x PBS and fixed in 4% PFA for 20 min on ice. Cells were stained with an appropriate Ghost Dye (TONBO biosciences) prior to fixation. Fixed cells were permeabilised in PBS with 5% donkey serum (Sigma) and 0.2% Triton X-100 for 30 minutes at 4˚C. Cells were incubated with primary antibodies in 1x PBS with 5% donkey serum and 0.1% Triton X100 overnight at 4˚C. The following day, cells were washed twice in 1x PBS and unconjugated antibodies were further incubated with secondary antibodies (Alexa Fluor conjugates) for 45 min. Antibody sources and concentrations are indicated in Supplementary Table 3.
Instrument	Cells were analysed using an LSRFortessa and FACSAria III (BD Biosciences).
Software	Data were analysed with the FACSDiva (BD Biosciences), FlowJo (BD Biosciences), and FCS Express 6 software (De Novo Software).
Cell population abundance	For stained samples, ten thousand events of live singlets were recorded. Based on unstained and isotype controls, gates were set to determine the number of positive signal events. The numbers of events were used to generate proportion of positive populations.
Gating strategy	Gates were set based on singlets, live/dead staining, unstained control, and isotype control to set positive gates of live single cells.

- [x] Tick this box to confirm that a figure exemplifying the gating strategy is provided in the Supplementary Information.
