# Supplementary material for: A 3D system to model human pancreas development and its reference single-cell transcriptome atlas identify signaling pathways required for progenitor expansion
Source: Nat Commun. 2021 May 25;12:3144. doi: 10.1038/s41467-021-23295-6 (PMC8149728; doi:10.1038/s41467-021-23295-6)
Supplement: Supplementary file 7 — Description of Additional Supplementary Files [file 41467_2021_23295_MOESM7_ESM.pdf]

Title: Supplementary Data 1.
Description: Top genes enriched in each cluster annotated for human fetal pancreas

Title: Supplementary Data 2.
Description: InterCom analysis of ligand-receptor pairs and downstream pathways

Title: Supplementary Data 3.
Description: Cell-cell communication networks inferred by state-of-the-art tools Cell-cell communication networks from each tool for each sample are provided together with a classification whether the interaction is unique or common to an interaction in the corresponding Intercom network.

Title: Supplementary Data 4.
Description: Genes differentially regulated upon expansion from Trott et al. (2017)”
